# DNA Methylation Modification Regulator-Mediated Molecular Clusters and Tumor Metabolic Characterization in Prostate Cancer

**DOI:** 10.1155/2021/2408637

**Published:** 2021-11-11

**Authors:** Yanlong Zhang, Xuezhi Liang, Liyun Zhang, Dongwen Wang

**Affiliations:** ^1^Department of Urology, First Hospital of Shanxi Medical University, Taiyuan, China; ^2^Shanxi Medical University, Taiyuan, China; ^3^Department of Rheumatology, Shanxi Bethune Hospital, Shanxi Academy of Medical Sciences, Taiyuan, China

## Abstract

**Background:**

An increasing number of studies have indicated a close link between DNA methylation and tumor metabolism. However, the overall influence of DNA methylation on tumor metabolic characteristics in prostate cancer (PCa) remains unclear.

**Methods:**

We first explored the subtypes of DNA methylation modification regulators and tumor metabolic features of 1,205 PCa samples using clustering analysis and gene set variation analysis based on the mRNA levels of DNA methylation modification regulators. A DNA methylation-related score (DMS) was calculated using principal component analysis and the DNA methylation modification-related gene signatures to quantify DNA methylation characteristics. We then performed a meta-analysis to identify the hazard ratio of DMS in the six cohorts. In addition, a nomogram was drawn using univariate and multivariate Cox analyses based on the DMS and clinical variables. Finally, a drug sensitivity analysis of the DMS was performed based on the genomics of drug sensitivity in cancer datasets.

**Results:**

Three PCa clusters showing different DNA methylation modification patterns and tumor metabolic features were identified. A DMS system was established to quantify the characteristics of DNA methylation modification. PCa samples showed a differential metabolic landscape between the high and low DMS groups. The prognostic value of the DMS and nomogram was independently validated in multiple cohorts. A high DMS was associated with increases in the tumor mutation burden, copy number variation, and microsatellite instability; high tumor heterogeneity; and poor prognosis. Finally, DMS was closely related to different types of antitumor treatment.

**Conclusion:**

Improving the understanding of tumor metabolism by characterizing DNA methylation modification patterns and using the DMS may help clinicians predict prognosis and aid in more personalized antitumor therapy strategies for PCa.

## 1. Introduction

DNA methylation is one of the earliest discovered and most widespread epigenetic modifications in cells [1]. Among the major forms of DNA methylation in humans is 5 mC, which occurs when DNA within CpG nucleotides is methylated at the fifth carbon atom of cytosine residues [2]. Prostate cancer (PCa) is the most common cancer in men [3]. Although major efforts have been devoted to improving the treatment of PCa, effective individualized therapeutic strategies require further analysis [4]. Several genes with essential roles in the initiation and progression of PCa have been reported to be regulated by promoter hypermethylation or hypomethylation. For example, cyclin-dependent kinase inhibitor 2A (*CDKN24*) is a suppressor gene that encodes the protein p16 and affects the cell cycle and hypermethylation in PCa [5, 6]. Decoy receptors 1 and 2, which are associated with cell apoptosis, maintain an abnormal DNA methylation status and promote the progression of PCa [7].

Cell development and multiplication are driven by energy metabolism. Based on the infinite proliferation ability of tumor cells, the initiation and growth of tumors are closely associated with the transformation of cell metabolic states [8]. Recent studies showed that the mechanism of PCa emergence is related to tumor metabolism, including citric acid and choline metabolism [9]. Some studies indicated that tumor metabolism associated with the androgen receptor (AR) leads to the occurrence and castration resistance of PCa [10, 11]. Thus, it may be possible to prevent the transformation to PCa by inhibiting these metabolic pathways. Studies are needed to explore tumor heterogeneity and the mechanism underlying metabolism in PCa.

Recent studies revealed that many key genes involved in tumor metabolism can be regulated by DNA methylation to promote tumor progression [12]. For example, DNA methylation can inhibit the expression of *MCT1* and impact glycolysis to render cells vulnerable to *MCT4* inhibition [13]. However, the overall impact of all DNA methylation regulators on tumor metabolism in PCa is unclear.

In this study, we collected 1,205 patients from six independent PCa cohorts and divided them into training and validation cohorts. We then performed unsupervised clustering based on DNA methylation modification (DMM) regulators and identified three DMM patterns in the training cohort. Because of the impact of upstream gene regulation based on the genomic context, we selected biomarkers that regulate DNA methylation rather than those that perform DNA methylation. Using single-sample gene set enrichment analysis (ssGSEA) based on metabolic pathways, we identified three DMM patterns and DMM-related gene clusters with different tumor metabolic characteristics and prognoses. Finally, we constructed a DNA methylation-related score (DMS) system to quantify DNA methylation characteristics. Through independent validation and meta-analysis, the DMS and nomogram based on the DMS can efficiently predict disease-free survival (DFS) in patients with PCa.

## 2. Materials and Methods

### 2.1. Collection of PCa Datasets

The workflow for this study is shown in Figure S1A. We collected data on PCa samples from the cBioPortal, Gene Expression Omnibus (GEO), and The Cancer Genome Atlas (TCGA) databases. First, we calculated the average mRNA expression data in multiple samples from the same patients. For prognostic analysis, we excluded patients without DFS information. We also collected six PCa cohorts (MSKCC, DKFZ, GSE116918, GSE54460, GSE70768, and TCGA) containing a total of 1,205 patients for calculation and analysis. For the TCGA prostate adenocarcinoma (PRAD) cohort, RNA sequencing data (fragments per kilobase transcript per million mapped reads [FPKM] values), clinical information, and DNA methylation data (450k methylation microarrays) were obtained from the Genomic Data Commons Data Portal (https://portal.gdc.cancer.gov). The MSKCC and DKFZ datasets were downloaded from cBioPortal for Cancer Genomics (http://www.cbioportal.org/); GSE54460, GSE70768, and GSE116918 were obtained from GEO (https://www.ncbi.nlm.nih.gov/geo/). The FPKM values were transformed into transcripts per kilobase million (TPM) values and log2(*n*+1) which was more consistent with the microarray data. Somatic mutation and copy number variation data of TCGA PRAD cohort were obtained from the University of California Santa Cruz (UCSC) Xena browser (https://xenabrowser.net). To validate our results in an independent cohort, we first combined the GSE54460, GSE70768, GSE116918, MSKCC, and DKFZ cohorts into one group which was designated as a meta (training) cohort to explore the characteristics of DNA methylation subtypes and construct the DMS. Using the R package sva, the “ComBat” algorithm was employed to remove batch effects (Figures S1B and S1C) [14]. We then used the TCGA PRAD cohort as the testing cohort to evaluate the prognostic value of the DMS and nomogram. All clinical information of the six PCa cohorts is presented in Table S1.

### 2.2. Unsupervised Clustering Analysis

To extract the characteristics of mRNA expression data and verify the DMM patterns and DMM-related gene clusters, we performed unsupervised clustering methods based on 20 DMM regulators and DMM-related genes in the meta cohort. The Partitioning Around Medoidclustering algorithm was applied and repeated 1,000 times using the R package ConsensusClusterPlus to ensure the stability of subtype analysis [15].

### 2.3. Single Sample Gene Set Enrichment Analysis

To quantify the activity level of each biological pathway, the R package GSVA was used to calculate the ssGSEA score for each sample [16]. Metabolic, immune-related, and tumor microenvironment-related gene signatures (epithelial-mesenchymal transition [EMT], extracellular matrix [ECM], and transforming growth factor-*β* [TGF-*β*]) used for ssGSEA were collected from the Molecular Signatures Database (MSigDB; http://www.gsea-msigdb.org/gsea/msigdb/) (Table S2) [17].

### 2.4. Differentially Expressed Gene Analysis

Differentially expressed genes (DEGs) between three DMM patterns with adjusted *P* value <0.001 were selected using the R package limma as DMM-related genes for further analysis [18].

### 2.5. Construction of DMS and Nomogram

We performed further statistical analysis of the prognostic DMM-related genes by performing univariate Cox regression analysis on 2259 DEGs, and 337 DMM-related genes associated with DFS were selected for further analysis. To quantify the characteristics of DNA methylation modification, we performed principal component analysis (PCA) of the meta cohort to generate a DMS system. PC1 and PC2 were extracted to calculate the DMS as shown in the equation below. The advantage of this approach is that it concentrates the score on the largest set of highly correlated (or unrelated) gene blocks in the set while downweighting the contribution of genes that are not tracked by other set members [19, 20].

DMS = Σ(PC1_*i*_−PC2_*i*_), where *i* is the expression of 337 prognostic DMM-related genes. The correlation between clinical variates, DMM regulators, DMS, and DFS of patients with PCa was analyzed by performing univariate analysis. The prognostic model and nomogram were constructed using multivariate Cox regression analysis. Kaplan–Meier (K-M) survival curves were used for prognostic analysis, and log-rank tests were performed to calculate *P* values. To test the precision of the risk model and nomogram, time-dependent receiver operating characteristic (ROC) analysis was performed using the R package survival ROC 1.0.3. An area under the ROC curve >0.60 indicates that the prediction ability of the model was meaningful, whereas a value of >0.75 indicates an outstanding predictive value of the model. The C-index was calculated using the R package pec. Decision curve analysis was performed using the R package ggDCA.

### 2.6. Gene Ontology and Kyoto Encyclopedia of Genes and Genomes Functional Enrichment Analysis

DEGs between the high and low DMS groups were ranked according to their logFC values. The top 1,000 DEGs were selected for subsequent analyses. Gene ontology (GO) and Kyoto Encyclopedia of Genes and Genomes (KEGG) analysis were performed to identify enriched GO and KEGG pathways of selected genes using the R package clusterProfiler with a cutoff of *P* value < 0.05 [21].

### 2.7. Statistical Analysis

Unpaired Student's *t*-test was used to compare two groups with normally distributed variables whereas the Mann–Whitney *U* test was used to compare two groups with nonnormally distributed variables. To compare the three groups, one-way analysis and Kruskal–Wallis tests of variance were used as parametric and nonparametric methods, respectively. Contingency table variables were analyzed using the chi-square test or Fisher's exact test. A combined analysis of the hazard ratio of DMS between the six cohorts was performed by meta-analysis (fixed-effect model). Fisher's exact test was used to calculate the difference for contingency table variables, and the correlation coefficient of two variables was obtained using Spearman's correlation analysis. Statistical significance was defined as a two-tailed *P* value < 0.05. All statistical analyses were performed using R software (version 3.6.3; The R Project for Statistical Computing, Vienna, Austria).

## 3. Results

### 3.1. Multiomics Landscape of DNA Methylation Regulators in PCa

**Figure 1:**
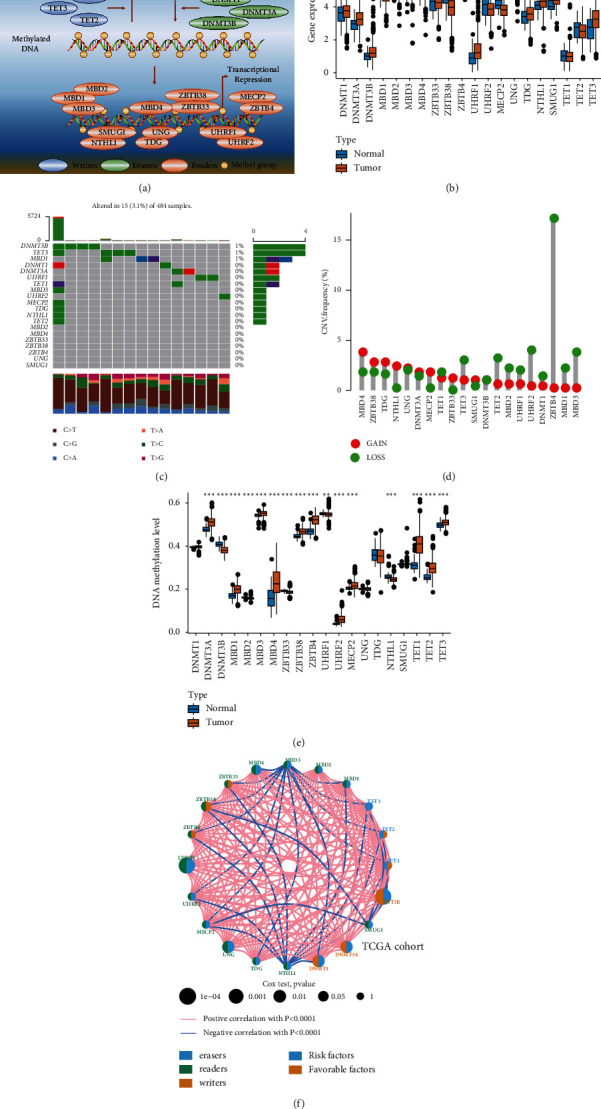
Multiomics landscape of DMM regulators in PCa. (a) Overview of the 20 DMM regulators and their major biological functions. (b) Boxplot shows the expression of 20 DMM regulators between tumor and normal tissues in the TCGAPRAD cohort. (c) The mutation frequency of 20 DMM regulators in TCGA PRAD cohort. Each column of the figure represents an individual patient. The upper bar plot represents TMB. The number on the right shows the mutation frequency of each regulator. The right bar plot indicates the proportion of each variant type. (d) The CNV frequency of DNA methylation regulators in TCGA PRAD cohort. (e) Boxplot shows the DNA methylation level of 20 DMM regulators between tumor and normal tissues in the TCGAPRAD cohort. (f) Correlations and prognosis of DMM regulators in PCa patients in TCGA PRAD cohort. The red line represents a positive correlation with *P* < 0.0001, and the blue line represents a negative correlation with *P* < 0.0001. The size of the node represents the *P* value of the log-rank test. Orange points represent favorable factors for DFS. Blue points represent risk factors for DFS. ^*∗*^*P* < 0.05, ^*∗∗*^*P* < 0.01, and ^*∗∗∗*^*P* < 0.001.

Figure 1(a) presents the 20 DMM regulators we collected from previous studies, including three erasers (TET1, TET2, and TET3), three writers (DNMT1, DNMT3A, and DNMT3B), and 14 readers (ZBTB33, ZBTB38, ZBTB4, UHRF1, UHRF2, MBD1, MBD2, MBD3, MBD4, UNG, MECP2, TDG, SMUG1, and NTHL1) [22–29]. We then performed differential expression analysis of these DMM regulators between paracancerous and PCa tissues in TCGA PRAD cohort. DNMT3A, DNMT3B, MBD3, UHRF1, TDG, NTHL1, SMUG1, and TET3 showed higher expression and MBD1, ZBTB38, ZBTB4, UHRF2, MECP2, and TDG showed lower expression in PCa samples than in normal prostate samples (Wilcoxon test: *P* < 0.05), as shown in Figure 1(b). To further evaluate these results, we compared the DNA mutation rate, copy number variation (CNV), and DNA methylation of all regulators. The overall mutation rate was relatively low in the PCa genome (Figure 1(c)), and the CNV rates of all regulators were less than 5%, except for that of ZBTB4 (Figure 1(d)). However, the DNA methylation levels of DNMT3A, DNMT3B, MBD1, MBD2, MBD3, MBD4, ZBTB33, ZBTB38, ZBTB4, UHRF1, UHRF2, MECP2, NTHL1, TET1, TET2, and TWT3 significantly differed between paracancerous and PCa tissues (Wilcoxon test: *P* < 0.05; Figure 1(e)). We also found that the methylation levels of ZBTB38, TDG, MBD1, SMUG1, DNMT3B, TET2, MBD2, UHRF1, and ZBTB4 were negatively correlated with the expression of these genes (Wilcoxon test: *P* < 0.05; Figure S2A and Table S3). Thus, DNA methylation may be an important driving factor leading to the aberrant expression of these DMM regulators.

### 3.2. Prognosis Value of DMM Regulators

We next examined the relationship between DMM regulators and DFS through prognostic analysis of PCa. First, we collected data from 1,205 patients with DFS and other clinical information from six independent cohorts (Table S1). We then divided these patients into a training cohort (meta cohort) to determine the characteristics of the DNA methylation and a testing cohort (TCGA PRAD cohort) to validate our conclusion. Univariate Cox regression and K-M survival analysis were used to analyze 20 DMM regulators in the meta and TCGA PRAD cohort, which showed that DNMT3B, UHRF1, UNG, DNMT1, DNMT3A, MBD4, ZBTB38, MBD3, SMUG1, UHRF2, TDG, NTHL1, TET1, ZBTB4, and ZBTB33 were closely related to the DFS of patients with PCa (log-rank test: *P* < 0.05; Table S4). Figures 1(f) and S2B present the correlation and prognostic value of these regulators in a network plot.

### 3.3. Identification of DMM Patterns

**Figure 2:**
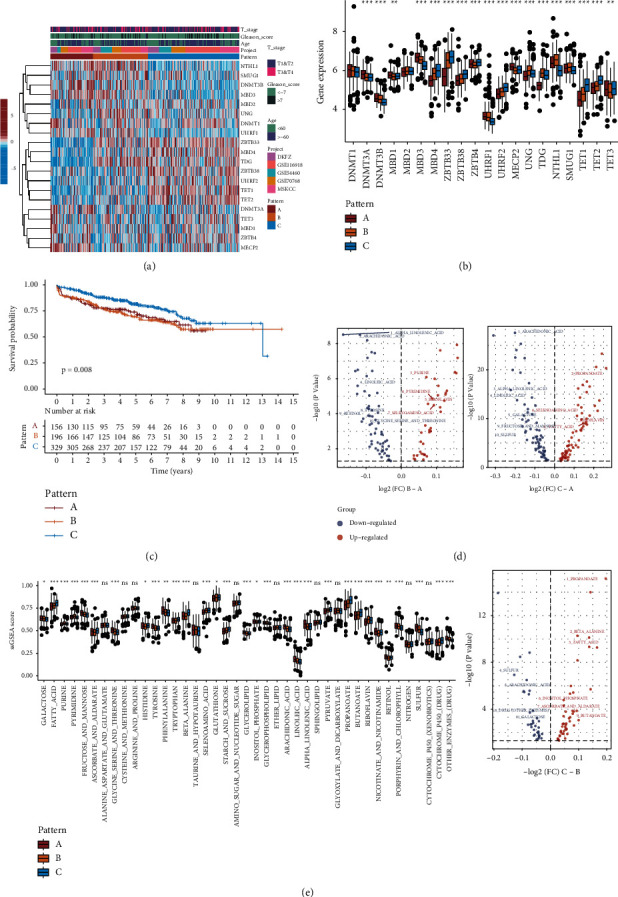
Consensus clustering of meta cohort. (a) Unsupervised clustering of 20 DMM regulators in the meta cohort. Red represents high expression, and blue represents low expression. The PCa cohorts and DNA methylation patterns were used as sample annotations. (b) Boxplot of 20 DMM regulators for three DMM patterns in the meta cohort. (c) Kaplan–Meier curves for the three DMM patterns of patients. The log-rank test showed an overall *P*=0.008. (d) The volcano plot of differential analysis based on ssGSEA and metabolic pathways between three DMM patterns in the meta cohort (only display top 10 -log10 (*P* value) metabolic pathways). (e) Boxplot of ssGSEA scores of 41 metabolic pathways for three DMM patterns in the meta cohort. ^*∗*^*P* < 0.05, ^*∗∗*^*P* < 0.01, and ^*∗∗∗*^*P* < 0.001.

As DMM regulators can impact the prognosis and tumor heterogeneity of patients with PCa, we further investigated potential DNA methylation modification regulator subtypes and features. Clustering analysis based on the expression level of 20 DMM regulators showed that the PCa meta cohort had the best clustering efficiency of the three DMM patterns (Figures 2(a) and S3A–S3H). Consequently, patients in the meta cohort were classified into three patterns: DMM pattern A (*n* = 156), DMM pattern B (*n* = 196), and DMM pattern C (*n* = 329). We first compared the expression levels of the regulators between the three DMM pattern groups and found significant differences in TET1, TET2, TET3, DNMT3A, DNMT3B, ZBTB33, ZBTB38, ZBTB4, UHRF1, UHRF2, MBD1, MBD3, MBD4, UNG, MECP2, TDG, SMUG1, and NTHL1 expression levels (Wilcoxon test: *P* < 0.05; Figure 2(b)). PCA based on the expression abundance of 20 DMM regulators showed that these three patterns were significantly separated, indicating that they were well-differentiated (Figure S3I). K-M survival curve analysis revealed that DMM pattern C was associated with a significantly longer DFS (log-rank test: *P* < 0.05; Figure 2(c)). To further identify the biological characteristics of each pattern, we quantified and compared the signaling pathways between the three DMM patterns and found that the activity levels of many metabolic pathways were significantly different between the three DMM patterns, suggesting that the metabolic status of PCa samples is regulated by DNA methylation (Wilcoxon test: *P* < 0.05; Figure 2(d) and Table S5). Finally, we performed a differential analysis of all metabolic pathways collected from KEGG and MSigBD between the three DMM patterns. The activity levels of 31/41 of these pathways were significantly different, further supporting our hypothesis (Wilcoxon test: *P* < 0.05; Figure 2(e)). These results indicate that DNA methylation plays an important role in the tumor metabolism-associated mechanism of PCa. In addition, we explored the correlation between the expression of DMM regulators and tumor metabolic ssGSEA scores; the heat map indicated that almost all DMM regulators were closely related to the tumor metabolic status in both the meta and TCGA PRAD cohort (Figure S4).

### 3.4. Features of DMM-Related Gene Clusters and Establishment of DMS

**Figure 3:**
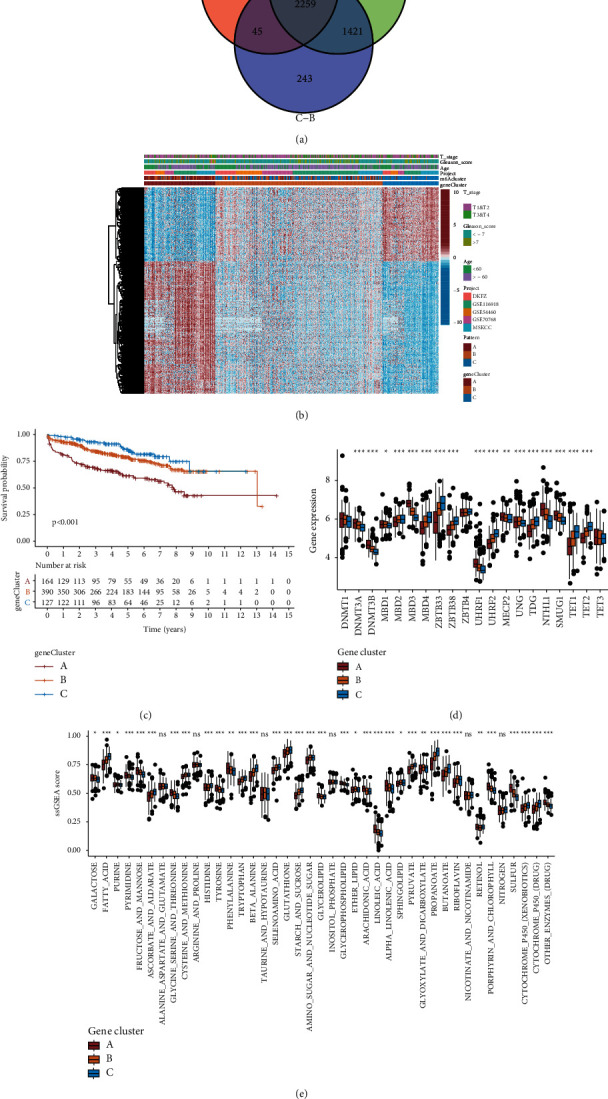
Transcriptomic and metabolic characteristics of the DMM-related gene cluster. (a) Venn diagram depicting 2259 differentially expressed genes in three DMM patterns. (b) Unsupervised clustering of 337 prognostic DMM-related genes in the meta cohort. Red represents high expression, and blue represents low expression. The PCa cohorts and DMM-related genes clusters were used as sample annotations. (c) Kaplan–Meier curves for the three DMM-related genes clusters of patients. The log-rank test showed an overall *P* < 0.001. (d) Boxplot of 20 DMM regulators for three DMM-related genes clusters in the meta cohort. (e) Boxplot of ssGSEA scores of 41 metabolic pathways for three DMM-related gene clusters in the meta cohort. ^*∗*^*P* < 0.05, ^*∗∗*^*P* < 0.01, and ^*∗∗∗*^*P* < 0.001.

To identify the heterogeneity and biomarkers of each pattern, we detected DEGs among DMM patterns through differential analysis. A total of 2,259 DNA DMM-related genes were selected from the meta cohort (*P* < 0.001; Figure 3(a) and Table S6). Univariate Cox regression analysis based on the meta-cohort confirmed that 337 DMM-related genes were associated with the DFS of patients with PCa (Table S7; univariate cox: *P* < 0.001). We also detected three DMM-related gene clusters based on the expression characteristics of these 337 genes using unsupervised cluster analysis (Figure 3(b) and Figures S5A–S5H).

Moreover, we compared the DFS of patients with PCa from different DMM-related gene clusters using K-M survival analysis. Patients in DMM-related gene cluster C had a longer DFS and better prognosis than those in DMM-related gene clusters A and B (log-rank test, *P* < 0.001; Figure 3(c)). We also found significant differences in the expression abundance of regulators, and 35/41 of the active levels of these pathways were significantly different between the three clusters (Wilcoxon test: *P* < 0.05; Figures 3(d) and 3(e)). The metabolic heat map of the PCa samples showed that each gene cluster had unique metabolic characteristics (Figure S6A). DMM-related gene cluster A showed elevated metabolic pathways, including pyrimidine, fructose and mannose, glycine, serine and threonine, tyrosine, glycerophospholipid, arachidonic acid, linoleic acid, alpha-linolenic acid, porphyrin and chlorophyll, sulfur, and other enzymes (drug) and exhibited the worst prognosis. Therefore, these pathways may be regulated by DNA methylation and promote PCa progression. In contrast, gene cluster C, with high active levels of fatty acid, ascorbate and aldarate, cysteine and methionine, histidine, tryptophan, beta-alanine, selenoamino acid, glutathione, starch and sucrose, pyruvate, propanoate, butanoate, cytochrome p450 (xenobiotics), and cytochrome p450 (drug) metabolic pathways showed the best prognosis, suggesting that these pathways suppress PCa progression. In addition, DMM-related gene cluster B is an intermediate metabolic subtype between DMM-related gene clusters A and C and had a medium prognosis. These findings demonstrate that DNA methylation is the source of tumor heterogeneity, leading to tumor metabolism disorders.

**Figure 4:**
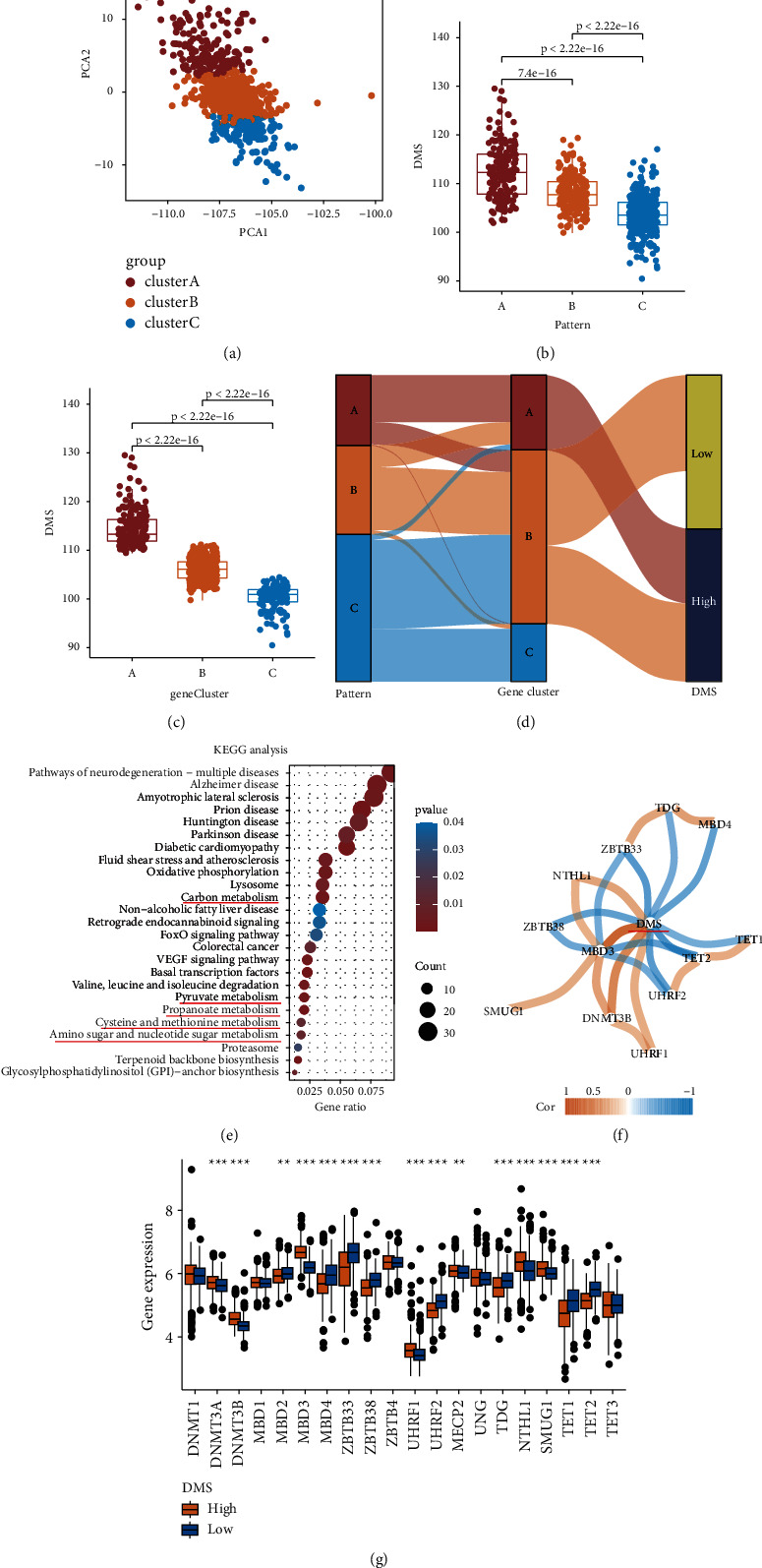
The relationship between DNA methylation and DMS. (a) Principal component analysis (PCA) based on 337 prognostic DMM-related gene expression. (b) Boxplot of DMS for three DMM patterns in the meta cohort. (c) Boxplot of DMS for three DMM-related gene clusters in the meta cohort. (d) Alluvial diagram of DMM patterns and DMM-related gene clusters distribution in groups with different DMS and survival outcomes (R represent recurrent and NR represent no recurrence). (e) KEGG analysis of DMS in meta cohorts (red line to mark the metabolic pathways). (f) The correlation network between 20 DMM regulators and DMS. (g) Boxplot of 20 DMM regulators for high and low DMS groups in meta cohort. ^*∗*^*P* < 0.05, ^*∗∗*^*P* < 0.01, and ^*∗∗∗*^*P* < 0.001.

To identify a quantitative biomarker of DNA methylation modification in PCa samples, we used the PCA algorithm to calculate the DMS, which is the value of PCA2 minus PCA1 from prognostic DMM-related genes (Figure 4(a) and Table S8). Differential analysis of DMS between different DMM patterns and DMM-related gene clusters indicated that DMS can describe the DNA methylation characteristics in PCa samples (Wilcoxon test: *P* < 0.05, Figures 4(b) and 4(c)). The distribution of patients in the meta cohort in the three patterns and three DMM-related gene clusters is shown in Figure 4(d). Finally, the GO and KEGG gene function enrichment analysis of DEGs between the high and low DMS groups divided by the median DMS (DMS = 106.32) in the meta cohort also indicated that DMS is related to carbon, pyruvate, propanoate, cysteine and methionine, and amino sugar and nucleotide sugar metabolisms (*P* < 0.05; Figure 4(e) and Table S9).

### 3.5. Relationship between DMS, DMM Regulators, DNA Methylation Level, and Metabolic Status of PCa Samples

To explore the relationship between DMS and DMM regulators, we calculated the coefficient between the DMS and DMM regulators and compared the expression levels of these regulators between the high and low DMS groups (Figures 4(f) and 4(g)). The results suggest that DMS is closely associated with the expression of TDG, MBD4, ZBTB33, NTHL1, ZBTB38, TET1, TET2, MBD3, SMUG1, DNMT3B, UHRF2, and UHRF1 (Spearman: correlation >0.3 and *P* < 0.05; Wilcoxon test: *P* < 0.05).

We also obtained TCGA PRAD 450 K data. First, we calculated the DMS of TCGA PRAD cohort samples and divided these samples into high and low DMS groups based on the median DMS (DMS = 72.10). Unsupervised clustering (single-center method) was performed based on the 500 genes showing the most variable DNA methylation levels in turn based on DNA methylation characteristics. Remarkably, the heat map indicated that the characteristics of DNA methylation were closely associated with the DMS (Figure S6B). We then compared the average DNA methylation levels of the top 500 genes between the high and low DMS groups and the coefficient between the average DNA methylation level and the DMS. The low DMS group had a higher DNA methylation level than the high DMS group, confirming that the DMS accurately reflected the DNA methylation status of PCa samples (Wilcoxon test: *P* < 0.05; Spearman correlation: *P* < 0.05; Figures S6C and S6D).

**Figure 5:**
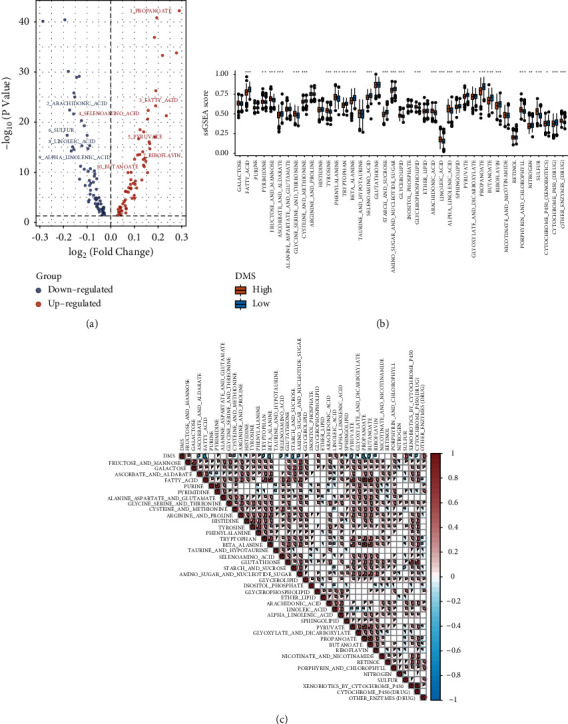
The relationship between DMS and tumor metabolism. (a) The volcano plot of differential analysis based on ssGSEA and KEGG pathways between low and high DMS groups in the meta cohort (only display top 10 -log10 (*P* value) metabolic pathways). (b) The heat map of the correlation between DMS and ssGSEA scores of 41 metabolic pathways in the meta cohort. (c) Boxplot of ssGSEA scores of 41 metabolic pathways for low and high DMS cohorts in the meta cohort. ^*∗*^*P* < 0.05, ^*∗∗*^*P* < 0.01, and ^*∗∗∗*^*P* < 0.001.

To verify the biological functions associated with DMS, we selected the significant differential ssGSEA scores of KEGG pathways and observed significant differences in the ssGSEA scores of metabolic pathways, including propanoate, arachidonic acid, fatty acid, sulfur, linoleic acid, riboflavin, alpha-linoleic acid, and 30/41 metabolic pathways (Wilcoxon test: *P* < 0.05; Figures 5(a) and 5(b), and Table S10). Therefore, we further explored the relationship between the DMS and tumor metabolic status. The activity levels of the metabolic pathways of fructose and mannose, pyrimidine, glycine, serine and threonine, tyrosine, phenylalanine, glycerolipid, glycerophospholipid, arachidonic acid, linoleic acid, alpha-linolenic acid, porphyrin and chlorophyll, sulfur, and other enzymes (drug) were positively correlated with the DMS and are considered to promote cancer factors. In contrast, the activity levels of ascorbate_and_aldarate, fatty_acid, cysteine_and_methionine, arginine_and_proline, histidine, tryptophan, beta_alanine, selenoamino_acid, glutathione, starch_and_sucrose, amino_sugar_and_nucleotide_sugar, sphingolipid, pyruvate, glyoxylate_and_dicarboxylate, propanoate, butanoate, riboflavin, xenobiotics_by_cytochrome_p450, and cytochrome_p450(drug) metabolic pathways were negatively correlated with the DMS and are considered to suppress cancer factors (Figure 5(c)).

### 3.6. Validation of Prognostic Value of DMS and Construction of Nomogram

**Figure 6:**
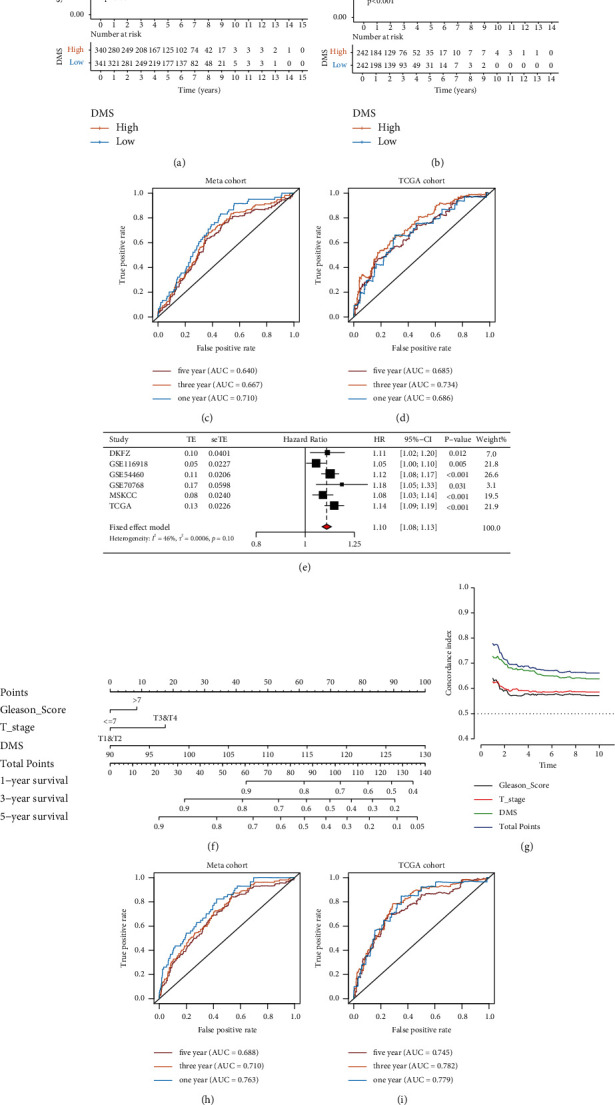
The prognostic value of DMS. (a) Kaplan–Meier curves for low and high DMS groups in the meta cohort. Log-rank test, *P* < 0.001. (b) Kaplan–Meier curves for low and high DMS group in the TCGA cohort. Log-rank test, *P* < 0.001. (c) The ROC analysis of DMS in the meta cohort. AUC = 0.710, 0.667, and 0.640 at 1, 3, and 5 years. (d) The ROC analysis of DMS in the TCGA cohort. AUC = 0.686, 0.734, and 0.685 at 1, 3, and 5 years. (e) The meta-analysis of the DMS's HR in six cohorts. (f) The nomogram based on DMS and clinical variates. (g) Concordance index of the nomogram in the meta cohorts. (h) The ROC analysis of nomogram in the meta (training) cohort. AUC = 0.763, 0.710, and 0.688 at 1, 3, and 5 years. (i) The ROC analysis of nomogram in the TCGA (validation) cohort. AUC = 0.779, 0.782, and 0.745 at 1, 3, and 5 years.

After determining the correlation between the DMS and metabolic status, we next verified the prognostic value of the DMS by performing KM survival curve analysis. The results suggested that patients in the low DMS group had a longer DFS time in the meta cohort (log-rank test: *P* < 0.001; Figure 6(a)). We then validated the prognostic value in the TCGA PRAD cohort (log-rank test: *P* < 0.001; Figure 6(b)). ROC analysis also indicated that the DMS predicted values in both the meta and TCGA PRAD cohorts and can predict DFS at 1, 3, and 5 years (Figures 6(c) and 6(d)). We validated the prognostic value of the DMS in each cohort in the meta cohort. The results were consistent with those of meta-analysis, and the low DMS group had a prolonged DFS in the GSE54460 (log-rank test: *P* < 0.001), GSE70768 (log-rank test: *P*=0.021), GSE116918 (log-rank test: *P*=0.013), DKFZ (log-rank test: *P*=0.159), and MSKCC (log-rank test: *P*=0.068) cohorts (Figures S7A–S7E). A possible reason that the *P* values of the DKFZ and MSKCC cohorts were greater than 0.05 is the small sample size. To determine the predictive value of the DMS in PCa, we performed univariate Cox analysis and meta-analysis to calculate the hazard ratio (HR) in six datasets (HR = 1.10; Figure 6(e)). The results also indicated that the DMS is a reliable prognostic marker.

This result was further examined by performing risk stratification analysis between the DMS and clinical factors, including age, Gleason score, and *T* stage, in both the meta and TCGA PRAD cohorts (Figures S8 and S9). The results confirmed that DMS is an independent prognostic biomarker with clinical factors and can accurately predict the DFS in patients with PCa. To improve the predictive value of DMS, we selected clinical variables with independent prognostic value to obtain a nomogram through univariate and multivariate Cox analysis in the meta cohort (*P* < 0.05; Figure 6(f) and Table S11). We used C-index calculation, ROC analysis, and decision curve analysis to assess the clinical significance of DMS, clinical variate, and nomogram. Superior results were observed for the DMS as a more accurate and reliable prognostic biomarker compared to clinical variates, whereas the nomogram had a better net benefit than the clinical variate or DMS-only models (Figures 6(g) and S10A–S10F). Furthermore, to independently validate the predictive value of the nomogram, we calculated the total points of each sample in the TCGA PRAD cohort and performed ROC and K-M survival curve analysis. The nomogram was found to predict the DFS of patients with PCa in both the meta and TCGA PRAD cohorts (Figures 6(h)–6(i), and Figures S10G and S10H).

### 3.7. Relationship between DMS, Tumor Mutation Burden, Microsatellite Instability, and Tumor Microenvironment

**Figure 7:**
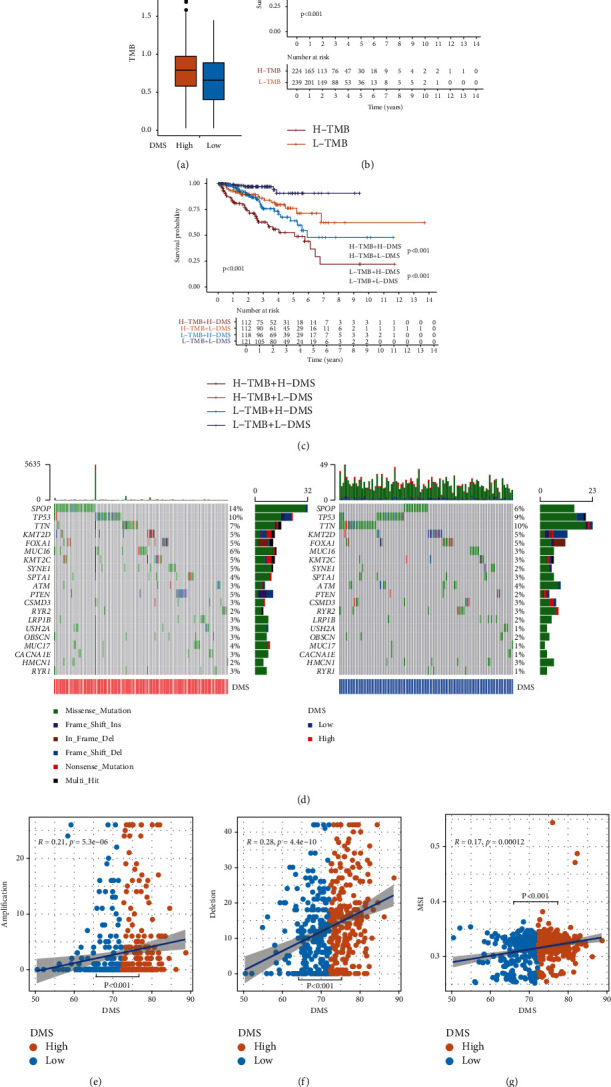
The relationship between DMS and somatic variants. (a) TMB difference in the high and low DMS groups in the TCGA cohort. Wilcoxon test, *P* < 0.001. (b) Kaplan–Meier curves for high and low TMB groups in the TCGA cohort. Log-rank test, *P* < 0.0011. (c) Kaplan–Meier curves for patients stratified by both TMB and DMS in the TCGA cohort. Log-rank test, *P* < 0.001. (d) The oncoPrint was constructed using a high DMS group on the left and a low DMS group on the right. Individual patients are represented in each column in the TCGA cohort. (e) The correlation between DMS and CNV (amplification) in the TCGA cohort. (f) The correlation between DMS and CNV (deletion) in the TCGA cohort. (g) The correlation between DMS and MSI in the TCGA cohort.

In previous studies, the tumor mutation burden (TMB) has been reported to reflect tumor heterogeneity [30]. Because nuclear excision repair showed functional enrichment for the DMS and to further examine the relationship between PCa tumor heterogeneity and DMS, we compared the TMB between the high and low DMS groups in the TCGA PRAD cohort. We found that tumors in the high DMS group had higher mutation counts than those in the low DMS group (Wilcoxon test: *P* < 0.001; Figure 7(a)). This further indicated that tumors in the high DMS group had greater heterogeneity than those in the low DMS group. We explored the relationship between the TMB, DMS, and DFS in prognosis by comparing the DFS between patients with a high and low TMB and found that patients with a high TMB had a shorter DFS than those with a low TMB (log-rank test: *P* < 0.001, Figure 7(b)). We further compared the DFS of high and low DMS groups in the high and low TMB groups. In the high or low TMB group, patients in the high DMS group had a shorter DFS than those in the low DMS group, suggesting that DMS is an independent prognostic factor for the TMB (log-rank test: *P* < 0.001; Figure 7(c)). We also generated a gene mutation landscape using a waterfall plot of the top 20 genes and found that the mutation rate of *SPOP* was higher and that of *TTN* was lower in the high DMS group (*P* < 0.05; Figure 7(d) and Table 1). Based on these results, DMS was clearly associated with DNA mutation in *SPOP* and *TTN*.

Copy number variations (CNVs) occurring upstream of genes regulate gene expression and influence tumor occurrence and development [31]. To explore whether this DNA element influences the DMS, we analyzed the number of amplifications and deletions as CNVs in DNA methylation regulator pattern-related prognostic genes. The number of CNV amplifications and deletions was higher in the high DMS group than in the low DMS group (Wilcoxon test: *P* < 0.001; Figures 7(e) and 7(f)), indicating that CNV is an important factor leading to DNA methylation disorders.

Microsatellite instability (MSI) is also a crucial indicator of genome instability associated with tumor heterogeneity [32]. We obtained the MSI data of each PRAD sample from a previous study and performed correlation analysis [33]. The results suggest that the high DMS group had a higher level of MSI compared to the low DMS group (Wilcoxon test: *P* < 0.001; Figure 7(g)), suggesting that MSI also interferes with DNA methylation.

The tumor microenvironment, including both stromal and immune cells, can impact the occurrence and progression of PCa. To explore the characteristics of PCa in the high/low DMS groups, we first calculated ssGSEA scores for EMT, ECM, and TGF-*β* using the corresponding gene sets (Table S2). Differential analysis of these ssGSEA scores indicated that high DMS tumors had higher ECM and lower TGF-*β* scores than low DMS tumors (Figure S11A). We then performed correlation analysis between immunologic ssGSEA scores and DMS, which revealed that the DMS was closely related to several immune pathways (Figure S11B). Therefore, DNA methylation may also be a crucial factor in immune disorders related to PCa.

### 3.8. Predictive Value of DMS in Antitumor Therapy

**Figure 8:**
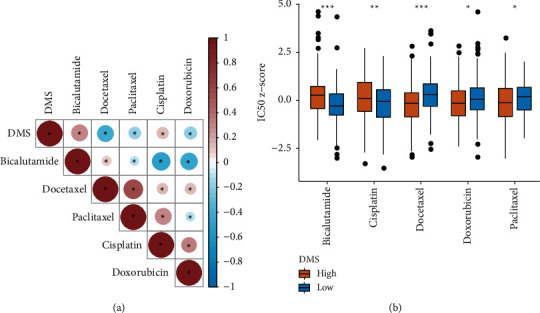
The drug-sensitive analysis of DMS. (a) The heat map of the correlation between DMS and IC50 of antitumor drugs in the meta cohort. (b) Boxplot of IC50 of antitumor drugs for low and high DMS cohorts in the meta cohort. ^*∗*^*P* < 0.05, ^*∗∗*^*P* < 0.01, and ^*∗∗∗*^*P* < 0.001.

For metastatic and recurrent prostate cancer, androgen deprivation therapy is the main treatment strategy and can significantly improve prognosis [34]. Based on this information, we first analyzed the relationship between the DMS and drug sensitivity of bicalutamide via meta-analysis. The results suggested a positive correlation between the DMS and half maximal inhibitory concentration (IC50) of bicalutamide, and tumor samples in the high DMS group had a higher IC50 on for bicalutamide compared to the low DMS group (Wilcoxon test, *P* < 0.001; Figures 8(a) and 8(b)). We then predicted the drug sensitivity to cisplatin, docetaxel, doxorubicin, and paclitaxel, which are common antitumor drugs used for PCa. The results shown in Figures 8(a) and 8(b) indicate that the DMS had predictive value for these drugs.

Immune therapy is the most widely used treatment for many cancers, and its effect is associated with the expression of checkpoint genes [35]. Although PCa is an immune desert tumor, some studies have suggested the usefulness of immunotherapy for PCa [36, 37]. Therefore, we compared the expression levels of checkpoint genes between the two groups. The high DMS group showed higher expression levels of *CD276*, *CD4*, *IL1A*, *LAG3A*, *PDCD1*, *TGFB1*, *CTLA4*, *CD8A*, *PRF1*, and *TNF* and lower expression levels of *CD274*, *CXCR4*, *CXCL10*, and *CXCL9* compared to the low DMS group (Wilcoxon test: *P* < 0.05; Figure S11C). The high expression level of checkpoint genes may explain the worse prognosis in the high DMS group, and patients in the high DMS group may benefit more from immune therapy than those in the low DMS group.

## 4. Discussion

With the development of molecular biology and oncology, increasing evidence has shown that DNA methylation can significantly influence tumor metabolism and cancer progression [38]. However, the overall spectrum of the regulatory patterns of DNA methylation and its influence on tumor metabolism in PCa remain unclear. It has been shown that the link between different DMM patterns and tumor metabolic status in PCa can promote the recognition of tumor pathology and be used to improve the efficiency of antitumor treatment.

In this study, we identified three DMM patterns with unique metabolic characteristics via clustering analysis of DMM regulator expression. PCa tissues showed significantly different metabolic statuses between DNA methylation regulator patterns. In addition, significantly different prognoses were observed between the three patterns.

The DEGs among these three DMM patterns were identified as DMM-related genes and may be directly or indirectly regulated by DNA methylation events. Similar to the DMM patterns, we found that three DMM-related gene clusters correlated with alterations in tumor metabolism were identified by these DMM-related genes. This is indicative of three different metabolic clusters in PCa. As pattern A and gene cluster A had the shortest DFS, the metabolic pathways activated in pattern A and gene cluster A, which are fructose_and_mannose, glycine_serine_and_threonine, tyrosine, glycerophospholipid, arachidonic_acid, linoleic_acid, alpha_linolenic_acid, porphyrin_and_chlorophyll, sulfur, and other_enzymes_(drug), may promote the progression of PCa, by altering metabolic pathways. Additionally, pattern C and gene cluster C were associated with the longest DFS and best prognosis, and their activated metabolic pathways fatty_acid, ascorbate_and_aldarate, cysteine_and_methionine, histidine, tryptophan, beta_alanine, selenoamino_acid, glutathione, starch_alanine, pyruvate, propanoate, butanoate, and cytochrome_p450(drug) may suppress PCa progression.

Considering the heterogeneity of DNA methylation modifications individually, it is necessary to quantify the DNA methylation modification spectrum of each sample. Therefore, we constructed a DMS system and validated it in multiple PCa cohorts. To confirm that the DMS is associated with DNA methylation characteristics, we conducted unsupervised clustering using DNA methylation levels of the top 500 genes with the greatest variation in PCa in the TCGA PRAD cohort. Unexpectedly, there was a close correlation between the DNA methylation status and DMS level (high/low DMS group). Taken together, these findings reveal an association between the transcriptome and DNA methylome and support that the DMS can represent the characteristics of DNA methylation.

Moreover, to validate the prognostic value of DMS, we performed hazard stratification and multivariate analysis between the DMS, TMB, Gleason score, and clinical factors in multiple PCa datasets. The results suggested that DMS is an independent prognostic factor and can help improve prognosis prediction in patients with PCa.

Finally, our results have clinical implications, as we established a DMS system that can describe DNA methylation characteristics. The prognostic value of the DMS system and nomogram based on clinical variables and DMS was validated in multiple PCa cohorts. The DMS can also be used to evaluate clinicopathological characteristics, such as the TMB, CNV, MSI status, and *SPOP* and *TTN* mutation in patients with PCa. In addition, DMS can predict the efficacy of castration, androgen deprivation therapy, and chemotherapy.

This study had some limitations. First, the reaction rate of the PCa antitumor therapy cohort was not accessible. The predictive value of the DMS for antitumor therapy in PCa must be further verified in further studies. Second, because of batch effects, the DMS cannot be directly compared between different cohorts without batch correction.

## 5. Conclusions

In summary, we explored three different metabolic subtypes based on the characteristics of DNA methylation and established a DMS system for PCa. DMS, which is associated with the tumor metabolic status, TMB, CNV, and MSI, is an efficient index for predicting DFS and therapeutic responsiveness and may help facilitate personalized antitumor therapy for patients with PCa.

## Figures and Tables

**Table 1:** Association of DMS with somatic variants.

Gene symbol	High DMS (%)	Low DMS (%)	*P* value
SPOP	44 (19%)	2 (1%)	*P* < 0.0001
TP53	12 (5%)	38 (16%)	*P*=0.0001
TTN	12 (5%)	29 (12%)	*P*=0.0084
CSMD1	1 (0%)	8 (3%)	*P*=0.0347
KMT2D	16 (7%)	7 (3%)	*P*=0.0554
FAT3	2 (1%)	9 (4%)	*P*=0.0625
LRP1B	4 (2%)	10 (10%)	*P*=0.1734
FOXA1	14 (6%)	8 (3%)	*P*=0.1952
SYNE1	5 (2%)	11 (5%)	*P*=0.2027
PTEN	5 (2%)	11 (5%)	*P*=0.2027
MUC17	3 (1%)	8 (3%)	*P*=0.2213
CSMD3	5 (2%)	9 (4)	*P*=0.4172
MUC16	7 (3%)	11 (5%)	*P*=0.4724
OBSCN	4 (2%)	7 (3%)	*P*=0.5442
USH2A	4 (2%)	7 (3%)	*P*=0.5442
HMCN1	7 (3%)	5 (2%)	*P*=0.5725
ATM	6 (3%)	9 (4%)	*P*=0.6016
SPTA1	7 (3%)	9 (4%)	*P*=0.8004
KMT2C	9 (4%)	10 (4%)	*P* > 0.9999
RYR2	6 (3%)	7 (3%)	*P* > 0.9999

## Data Availability

The datasets used and/or analyzed during the current study are available from TCGA, GEO, cBio, and GDSC databases.

## Abbreviations

AR: Androgen receptor
AUC: Area under the ROC curve
CNV: Copy number variation
DCA: Decision curve analysis
DEG: Differentially expressed gene
DFS: Disease-free survival
DMM: DNA methylation modification
DMS: DNA methylation-related score
ECM: Extracellular matrix
EMT: Epithelial-mesenchymal transition
FPKM: Fragments per kilobase transcript per million mapped reads
GEO: Gene Expression Omnibus
GO: Gene ontology
HR: Hazard ratio
IC50: Half maximal inhibitory concentration
KEGG: Kyoto Encyclopedia of Genes and Genomes
K-M: Kaplan–Meier
MSI: Microsatellite instability
MSigDB: Molecular signatures database
PCa: Prostate cancer
PCA: Principal component analysis
PRAD: Prostate adenocarcinoma
ROC: Receiver operating characteristic
ssGSEA: Single sample gene set enrichment analysis
TCGA: Cancer Genome Atlas
TGF-*β*: Transforming growth factor-beta
TMB: Tumor mutation burden
TME: Tumor microenvironment
TPM: Transcripts per kilobase million
UCSU: University of California Santa Cruz.

## Supplementary Materials

Supplementary Materials Supplemental Figure 1: (A) overview of study design. (B) Principal component analysis (PCA) of mRNA expression data of sample from five cohorts before batch correction. (C) Principal component analysis (PCA) of mRNA expression data of sample from five cohorts after batch correction. Supplemental Figure 2: (A) the volcano plot for correlation analysis between DNA methylation and mRNA expression of 20 DMM regulators. (B) Correlations and prognosis of DMM regulators in PCa patients in TCGA PRAD cohort. The red line represents a positive correlation with *P* < 0.0001, and the blue line represents a negative correlation with *P* < 0.0001. The size of the node represents the *p* value of the log-rank test. Orange points represent favorable factors for DFS. Blue points represent risk factors for DFS. ^*∗*^*P* < 0.05, ^*∗∗*^*P* < 0.01, and ^*∗∗∗*^*P* < 0.001. Supplemental Figure 3: (A–H) consensus matrixes of PCa samples for each *k* (*k* = 2–7) from meta cohort based on expression abundance of 20 DMM regulators, displaying the clustering stability using 1000 iterations of hierarchical clustering. (H) Principal component analysis (PCA) of meta cohort based on expression abundance of 20 DMM regulators. Supplemental Figure 4: the correlation between ssGSEA score of 41 metabolic pathways and expression abundance of 20 DMM regulators in meta cohort and TCGA cohort. Supplemental Figure 5: (A–H) consensus matrixes of PCa samples for each *k* (*k* = 2–7) from meta cohort based on expression abundance of 337 DNA methylation-related prognostic genes, displaying the clustering stability using 1000 iterations of hierarchical clustering. Supplemental Figure 6: the relationship between DMS, tumor metabolism, and DNA methylation statue. (A) The heat map of the active level of metabolic pathways for three DNA methylation gene clusters in meta cohort. (B) The heat map of DNA methylation level (variance top 500 genes) for low and high DMS group in TCGA cohort. (C) Boxplot of average DNA methylation level (variance top 500 genes) for low and high DMS groups in the TCGA cohort, *P* < 0.001. (D) The correlation between DMS and average DNA methylation level (variance top 500 genes) in TCGA cohort. Supplemental Figure 7: Kaplan–Meier curves for the low/high DMS groups in DKFZ cohort (A), GSE54460 cohort (B), GSE70768 (C), GSE116918 (D), and MSKCC (*E*). Supplemental Figure 8: hazard stratification analysis in meta cohort. age ≥ 60 (A), <60 (B), Gleason score>7 (C), Gleason score ≤ 7 (D), T1-T2 (E), and T3-T4 (F). Supplemental Figure 9: hazard stratification analysis in TCGA cohort. Age ≥ 60 (A), <60 (B), Gleason score>7 (C), Gleason score ≤ 7 (D), T1-T2 (E), and T3-T4 (F). Supplemental Figure 10: the predict value of nomogram. The ROC analysis for nomogram in 1 year (A), 3 years (B), and 5 years (C). The DCA for nomogram in 1 year (D), 3 years (E), and 5 years (F). Kaplan–Meier curves for the low/high total points groups in meta (training) (G) cohort and TCGA (validation) (H) cohort. Supplemental Figure 11: the relationship between DMS and TME. (A) Boxplot of ssGSEA score of EMT, ECM, and TGF-*β* for low and high DMS groups in the meta cohort. (B) The heat map of the correlation between DMS and ssGSEA scores of immune pathways in meta cohort. (C) Boxplot of ssGSEA score of immune pathways for low and high DMS groups in the meta cohort. ^*∗*^*P* < 0.05, ^*∗∗*^*P* < 0.01, and ^*∗∗∗*^*P* < 0.001. Supplemental Table 1: clinical information of PCa patients from six cohorts. Supplemental Table 2: gene sets analyzed in the article. Supplemental Table 3: correlation analysis between DNA methylation and mRNA expression of 20 DMM regulators. Supplemental Table 4: the univariate cox analysis of 20 DMM regulators in meta cohort and TCGA cohort. Supplemental Table 5: differential analysis of ssGSEA of KEGG pathways for three DMM patterns. Supplemental Table 6: DEGs between three DMM patterns. Supplemental Table 7: the univariate cox analysis of 337 prognostic DMM-related gene expression in meta cohort. Supplemental Table 8: PCA and PCA coefficient to calculated DMS. Supplemental Table 9: GO/KEGG analysis of DEGs between low and high DMS groups in meta cohort. Supplemental Table 10: differential analysis of ssGSEA of KEGG pathways for low and high DMS groups. Supplemental Table 11: the univariate and multivariate cox analysis of DMS and clinical variates.
