# Supplementary material for: DNA Methylation Modification Regulator-Mediated Molecular Clusters and Tumor Metabolic Characterization in Prostate Cancer
Source: J Oncol. 2021 Nov 11;2021:2408637. doi: 10.1155/2021/2408637 (PMC8601836; doi:10.1155/2021/2408637)
Supplement: Supplementary Materials — Supplemental Figure 1: (A) overview of study design. (B) Principal component analysis (PCA) of mRNA expression data of sample from five cohorts before batch correction. (C) Principal component analysis (PCA) of mRNA expression data of sample from five cohorts after batch correction. Supplemental Figure 2: (A) the volcano plot for correlation analysis between DNA methylation and mRNA expression of 20 DMM regulators. (B) Correlations and prognosis of DMM regulators in PCa patients in TCGA PRAD cohort. The red line represents a positive correlation with P < 0.0001, and the blue line represents a negative correlation with P < 0.0001. The size of the node represents the p value of the log-rank test. Orange points represent favorable factors for DFS. Blue points represent risk factors for DFS. ∗P < 0.05, ∗∗P < 0.01, and ∗∗∗P < 0.001. Supplemental Figure 3: (A–H) consensus matrixes of PCa samples for each k (k = 2–7) from meta cohort based on expression abundance of 20 DMM regulators, displaying the clustering stability using 1000 iterations of hierarchical clustering. (H) Principal component analysis (PCA) of meta cohort based on expression abundance of 20 DMM regulators. Supplemental Figure 4: the correlation between ssGSEA score of 41 metabolic pathways and expression abundance of 20 DMM regulators in meta cohort and TCGA cohort. Supplemental Figure 5: (A–H) consensus matrixes of PCa samples for each k (k = 2–7) from meta cohort based on expression abundance of 337 DNA methylation-related prognostic genes, displaying the clustering stability using 1000 iterations of hierarchical clustering. Supplemental Figure 6: the relationship between DMS, tumor metabolism, and DNA methylation statue. (A) The heat map of the active level of metabolic pathways for three DNA methylation gene clusters in meta cohort. (B) The heat map of DNA methylation level (variance top 500 genes) for low and high DMS group in TCGA cohort. (C) Boxplot of average DNA methylation level (variance top 500 [file 2408637.f1.zip › Supplemental Figure.zip!/Supplemental Figure/Supplemental Figure 1.pdf]

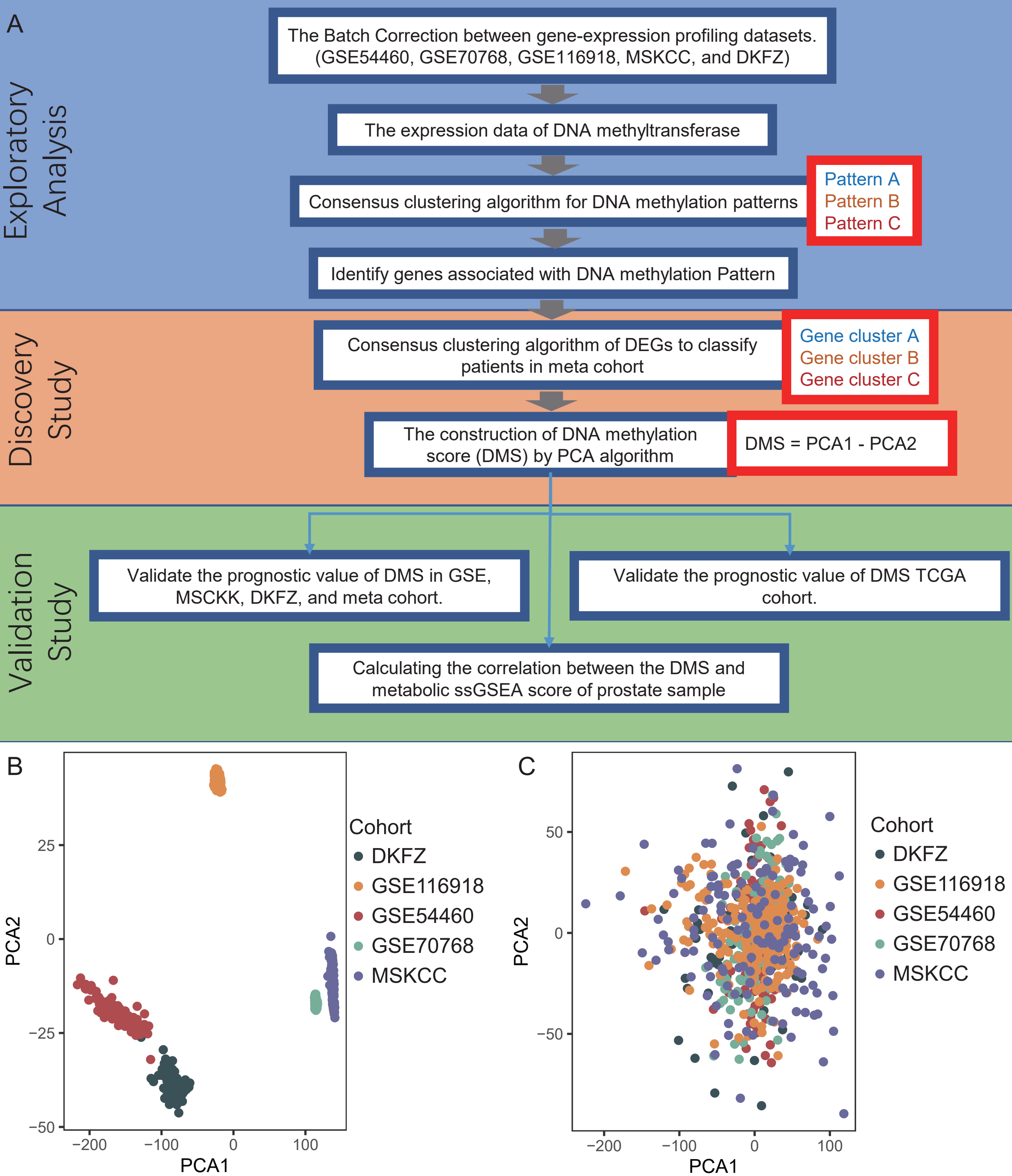

A
Exploratory Analysis
The Batch Correction between gene-expression profiling datasets. (GSE54460, GSE70768, GSE116918, MSKCC, and DKFZ)
The expression data of DNA methyltransferase
Consensus clustering algorithm for DNA methylation patterns
Pattern A
Pattern B
Pattern C
Identify genes associated with DNA methylation Pattern
Discovery Study
Consensus clustering algorithm of DEGs to classify patients in meta cohort
Gene cluster A
Gene cluster B
Gene cluster C
The construction of DNA methylation score (DMS) by PCA algorithm
DMS = PCA1 - PCA2
Validation Study
Validate the prognostic value of DMS in GSE, MSCKK, DKFZ, and meta cohort.
Validate the prognostic value of DMS TCGA cohort.
Calculating the correlation between the DMS and metabolic ssGSEA score of prostate sample
B
PCA2
PCA1
25
0
−25
−50
−200
−100
0
100
Cohort
DKFZ
GSE116918
GSE54460
GSE70768
MSKCC
C
PCA2
PCA1
50
0
−50
−200
−100
0
100
Cohort
DKFZ
GSE116918
GSE54460
GSE70768
MSKCC
