# Supplementary material for: DNA Methylation Modification Regulator-Mediated Molecular Clusters and Tumor Metabolic Characterization in Prostate Cancer
Source: J Oncol. 2021 Nov 11;2021:2408637. doi: 10.1155/2021/2408637 (PMC8601836; doi:10.1155/2021/2408637)
Supplement: Supplementary Materials — Supplemental Figure 1: (A) overview of study design. (B) Principal component analysis (PCA) of mRNA expression data of sample from five cohorts before batch correction. (C) Principal component analysis (PCA) of mRNA expression data of sample from five cohorts after batch correction. Supplemental Figure 2: (A) the volcano plot for correlation analysis between DNA methylation and mRNA expression of 20 DMM regulators. (B) Correlations and prognosis of DMM regulators in PCa patients in TCGA PRAD cohort. The red line represents a positive correlation with P < 0.0001, and the blue line represents a negative correlation with P < 0.0001. The size of the node represents the p value of the log-rank test. Orange points represent favorable factors for DFS. Blue points represent risk factors for DFS. ∗P < 0.05, ∗∗P < 0.01, and ∗∗∗P < 0.001. Supplemental Figure 3: (A–H) consensus matrixes of PCa samples for each k (k = 2–7) from meta cohort based on expression abundance of 20 DMM regulators, displaying the clustering stability using 1000 iterations of hierarchical clustering. (H) Principal component analysis (PCA) of meta cohort based on expression abundance of 20 DMM regulators. Supplemental Figure 4: the correlation between ssGSEA score of 41 metabolic pathways and expression abundance of 20 DMM regulators in meta cohort and TCGA cohort. Supplemental Figure 5: (A–H) consensus matrixes of PCa samples for each k (k = 2–7) from meta cohort based on expression abundance of 337 DNA methylation-related prognostic genes, displaying the clustering stability using 1000 iterations of hierarchical clustering. Supplemental Figure 6: the relationship between DMS, tumor metabolism, and DNA methylation statue. (A) The heat map of the active level of metabolic pathways for three DNA methylation gene clusters in meta cohort. (B) The heat map of DNA methylation level (variance top 500 genes) for low and high DMS group in TCGA cohort. (C) Boxplot of average DNA methylation level (variance top 500 [file 2408637.f1.zip › Supplemental Figure.zip!/Supplemental Figure/Supplemental Figure 10.pdf]

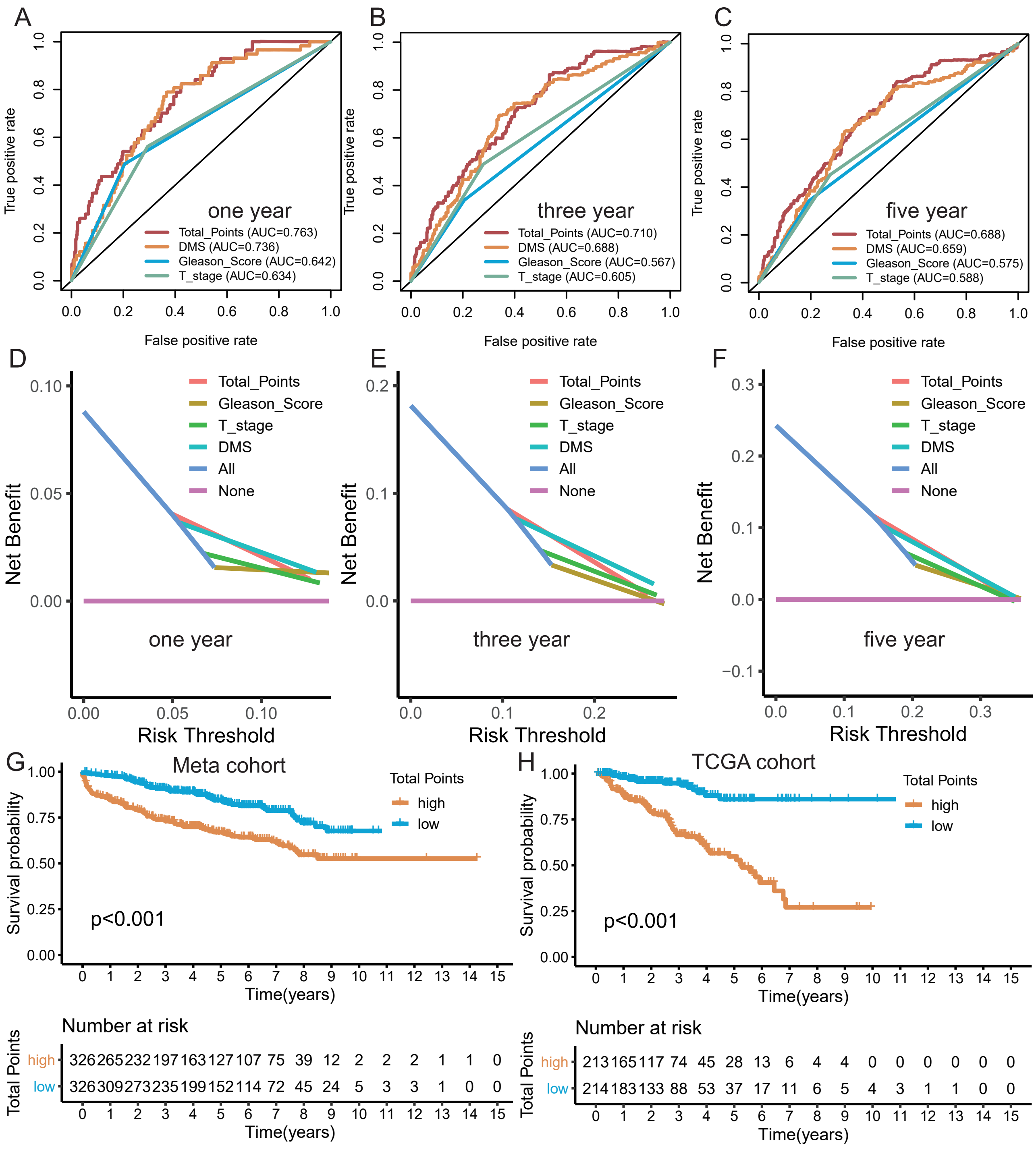
A
True positive rate
False positive rate
one year
Total_Points (AUC=0.763)
DMS (AUC=0.736)
Gleason_Score (AUC=0.642)
T_stage (AUC=0.634)
B
three year
Total_Points (AUC=0.710)
DMS (AUC=0.688)
Gleason_Score (AUC=0.567)
T_stage (AUC=0.605)
C
five year
Total_Points (AUC=0.688)
DMS (AUC=0.659)
Gleason_Score (AUC=0.575)
T_stage (AUC=0.588)
D
Net Benefit
Risk Threshold
Total_Points
Gleason_Score
T_stage
DMS
All
None
one year
E
three year
F
five year
G
Meta cohort
Survival probability
Time(years)
Total Points
high
low
p<0.001
Number at risk
high 326 265 232 197 163 127 107 75 39 12 2 2 2 1 1 0
low 326 309 273 235 199 152 114 72 45 24 5 3 3 1 0 0
H
TCGA cohort
p<0.001
high 213 165 117 74 45 28 13 6 4 4 0 0 0 0 0 0
low 214 183 133 88 53 37 17 11 6 5 4 3 1 1 0 0
