# Supplementary material for: DNA Methylation Modification Regulator-Mediated Molecular Clusters and Tumor Metabolic Characterization in Prostate Cancer
Source: J Oncol. 2021 Nov 11;2021:2408637. doi: 10.1155/2021/2408637 (PMC8601836; doi:10.1155/2021/2408637)
Supplement: Supplementary Materials — Supplemental Figure 1: (A) overview of study design. (B) Principal component analysis (PCA) of mRNA expression data of sample from five cohorts before batch correction. (C) Principal component analysis (PCA) of mRNA expression data of sample from five cohorts after batch correction. Supplemental Figure 2: (A) the volcano plot for correlation analysis between DNA methylation and mRNA expression of 20 DMM regulators. (B) Correlations and prognosis of DMM regulators in PCa patients in TCGA PRAD cohort. The red line represents a positive correlation with P < 0.0001, and the blue line represents a negative correlation with P < 0.0001. The size of the node represents the p value of the log-rank test. Orange points represent favorable factors for DFS. Blue points represent risk factors for DFS. ∗P < 0.05, ∗∗P < 0.01, and ∗∗∗P < 0.001. Supplemental Figure 3: (A–H) consensus matrixes of PCa samples for each k (k = 2–7) from meta cohort based on expression abundance of 20 DMM regulators, displaying the clustering stability using 1000 iterations of hierarchical clustering. (H) Principal component analysis (PCA) of meta cohort based on expression abundance of 20 DMM regulators. Supplemental Figure 4: the correlation between ssGSEA score of 41 metabolic pathways and expression abundance of 20 DMM regulators in meta cohort and TCGA cohort. Supplemental Figure 5: (A–H) consensus matrixes of PCa samples for each k (k = 2–7) from meta cohort based on expression abundance of 337 DNA methylation-related prognostic genes, displaying the clustering stability using 1000 iterations of hierarchical clustering. Supplemental Figure 6: the relationship between DMS, tumor metabolism, and DNA methylation statue. (A) The heat map of the active level of metabolic pathways for three DNA methylation gene clusters in meta cohort. (B) The heat map of DNA methylation level (variance top 500 genes) for low and high DMS group in TCGA cohort. (C) Boxplot of average DNA methylation level (variance top 500 [file 2408637.f1.zip › Supplemental Figure.zip!/Supplemental Figure/Supplemental Figure 11.pdf]

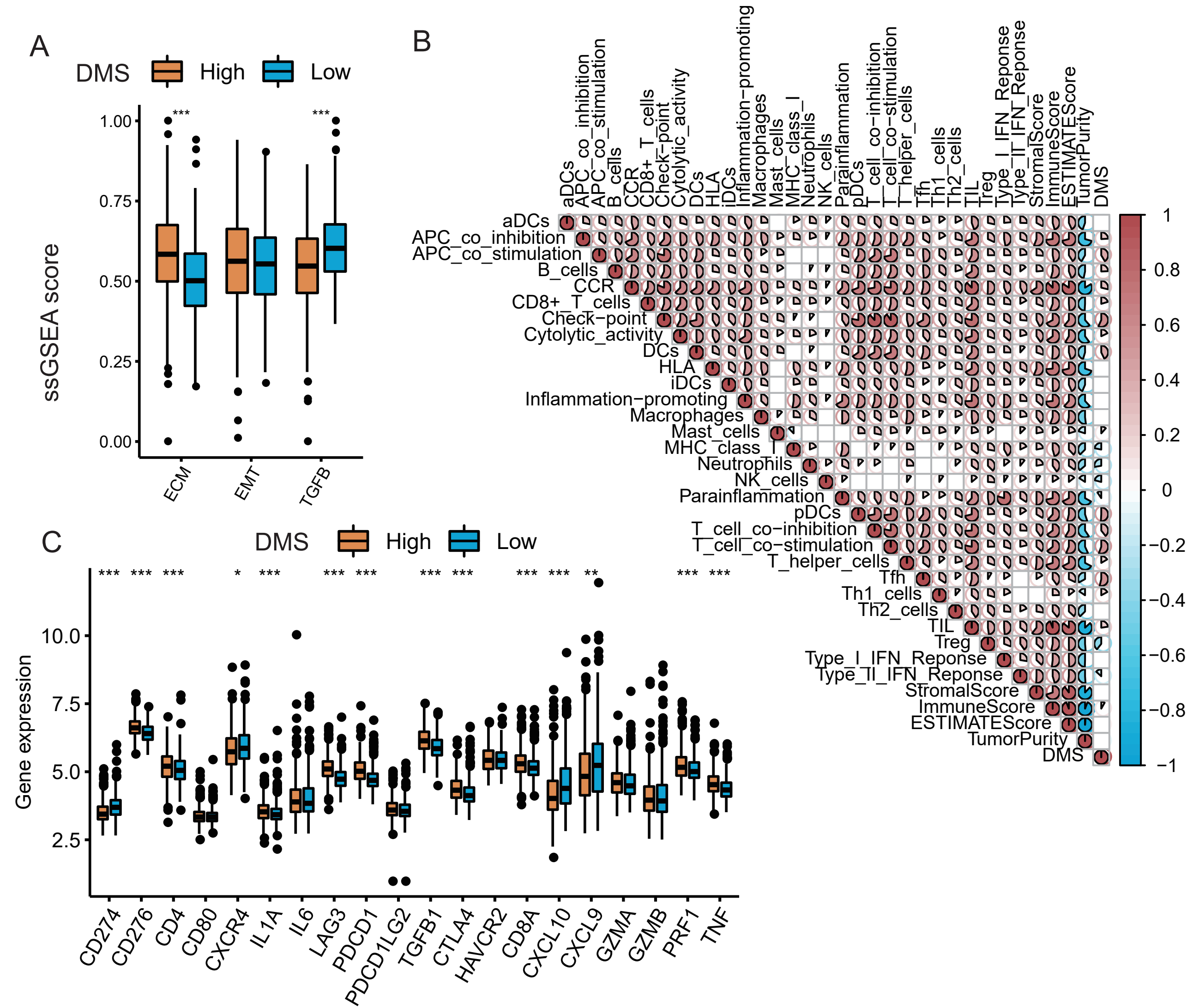

A
DMS High Low
ssGSEA score
1.00
0.75
0.50
0.25
0.00
***
***
ECM
EMT
TGFB
B
aDCs
APC_co_inhibition
APC_co_stimulation
B_cells
CCR
CD8+_T_cells
Check−point
Cytolytic_activity
DCs
HLA
iDCs
Inflammation−promoting
Macrophages
Mast_cells
MHC_class_I
Neutrophils
NK_cells
Parainflammation
pDCs
T_cell_co−inhibition
T_cell_co−stimulation
T_helper_cells
Tfh
Th1_cells
Th2_cells
TIL
Treg
Type_I_IFN_Reponse
Type_II_IFN_Reponse
StromalScore
ImmuneScore
ESTIMATEScore
TumorPurity
DMS
1
0.8
0.6
0.4
0.2
0
−0.2
−0.4
−0.6
−0.8
−1
C
DMS High Low
Gene expression
10.0
7.5
5.0
2.5
*** *** *** * *** *** *** *** *** *** *** ** *** ***
CD274
CD276
CD4
CD80
CXCR4
IL1A
IL6
LAG3
PDCD1
PDCD1LG2
TGFB1
CTLA4
HAVCR2
CD8A
CXCL10
CXCL9
GZMA
GZMB
PRF1
TNF
