# Supplementary material for: DNA Methylation Modification Regulator-Mediated Molecular Clusters and Tumor Metabolic Characterization in Prostate Cancer
Source: J Oncol. 2021 Nov 11;2021:2408637. doi: 10.1155/2021/2408637 (PMC8601836; doi:10.1155/2021/2408637)
Supplement: Supplementary Materials — Supplemental Figure 1: (A) overview of study design. (B) Principal component analysis (PCA) of mRNA expression data of sample from five cohorts before batch correction. (C) Principal component analysis (PCA) of mRNA expression data of sample from five cohorts after batch correction. Supplemental Figure 2: (A) the volcano plot for correlation analysis between DNA methylation and mRNA expression of 20 DMM regulators. (B) Correlations and prognosis of DMM regulators in PCa patients in TCGA PRAD cohort. The red line represents a positive correlation with P < 0.0001, and the blue line represents a negative correlation with P < 0.0001. The size of the node represents the p value of the log-rank test. Orange points represent favorable factors for DFS. Blue points represent risk factors for DFS. ∗P < 0.05, ∗∗P < 0.01, and ∗∗∗P < 0.001. Supplemental Figure 3: (A–H) consensus matrixes of PCa samples for each k (k = 2–7) from meta cohort based on expression abundance of 20 DMM regulators, displaying the clustering stability using 1000 iterations of hierarchical clustering. (H) Principal component analysis (PCA) of meta cohort based on expression abundance of 20 DMM regulators. Supplemental Figure 4: the correlation between ssGSEA score of 41 metabolic pathways and expression abundance of 20 DMM regulators in meta cohort and TCGA cohort. Supplemental Figure 5: (A–H) consensus matrixes of PCa samples for each k (k = 2–7) from meta cohort based on expression abundance of 337 DNA methylation-related prognostic genes, displaying the clustering stability using 1000 iterations of hierarchical clustering. Supplemental Figure 6: the relationship between DMS, tumor metabolism, and DNA methylation statue. (A) The heat map of the active level of metabolic pathways for three DNA methylation gene clusters in meta cohort. (B) The heat map of DNA methylation level (variance top 500 genes) for low and high DMS group in TCGA cohort. (C) Boxplot of average DNA methylation level (variance top 500 [file 2408637.f1.zip › Supplemental Figure.zip!/Supplemental Figure/Supplemental Figure 2.pdf]

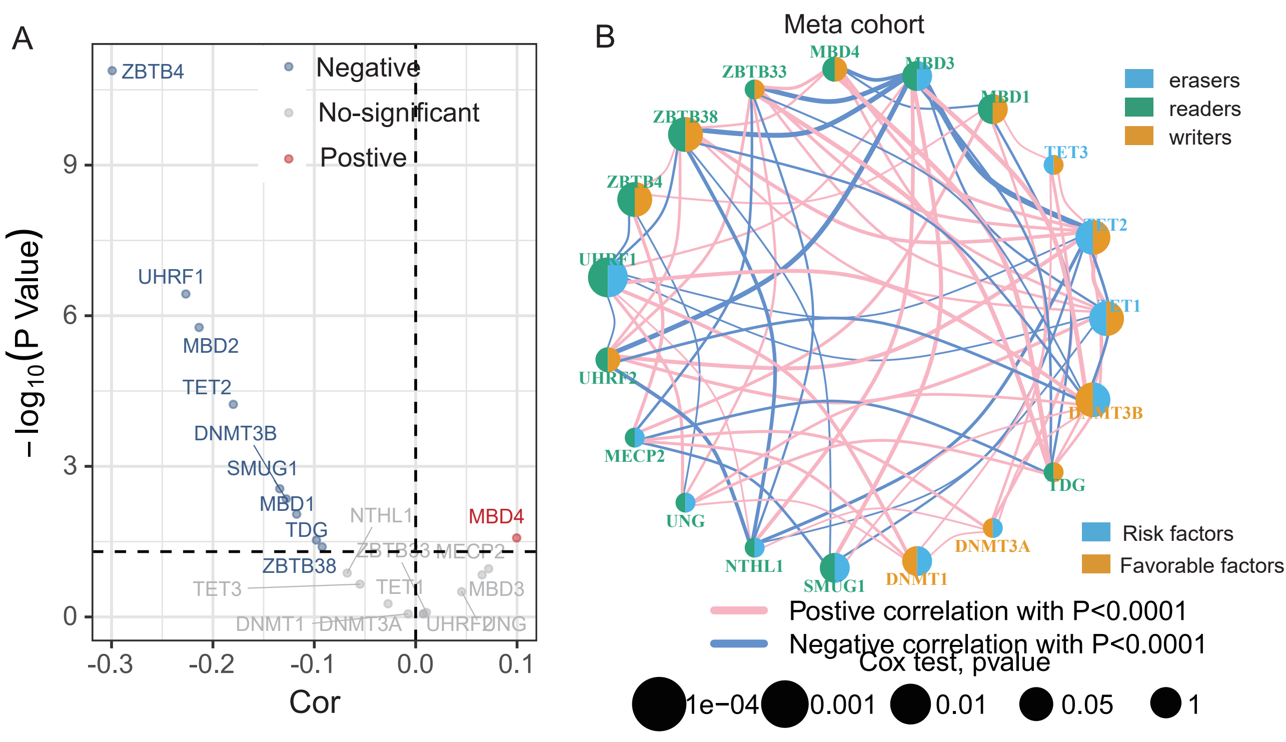
A
ZBTB4
Negative
No-significant
Postive
UHRF1
MBD2
TET2
DNMT3B
SMUG1
MBD1
TDG
NTHL1
MBD4
ZBTB38
ZBTB33
MECP2
TET3
TET1
MBD3
DNMT1
DNMT3A
UHRF2
UNG
9
6
3
0
-0.3
-0.2
-0.1
0.0
0.1
Cor
$-\log_{10}(\text{P Value})$
B
Meta cohort
MBD4
MBD3
ZBTB33
MBD1
ZBTB38
TET3
ZBTB4
TET2
UHRF1
TET1
UHRF2
DNMT3B
MECP2
TDG
UNG
DNMT3A
NTHL1
SMUG1
DNMT1
erasers
readers
writers
Risk factors
Favorable factors
Postive correlation with P<0.0001
Negative correlation with P<0.0001
Cox test, pvalue
1e−04
0.001
0.01
0.05
1
