# Supplementary material for: DNA Methylation Modification Regulator-Mediated Molecular Clusters and Tumor Metabolic Characterization in Prostate Cancer
Source: J Oncol. 2021 Nov 11;2021:2408637. doi: 10.1155/2021/2408637 (PMC8601836; doi:10.1155/2021/2408637)
Supplement: Supplementary Materials — Supplemental Figure 1: (A) overview of study design. (B) Principal component analysis (PCA) of mRNA expression data of sample from five cohorts before batch correction. (C) Principal component analysis (PCA) of mRNA expression data of sample from five cohorts after batch correction. Supplemental Figure 2: (A) the volcano plot for correlation analysis between DNA methylation and mRNA expression of 20 DMM regulators. (B) Correlations and prognosis of DMM regulators in PCa patients in TCGA PRAD cohort. The red line represents a positive correlation with P < 0.0001, and the blue line represents a negative correlation with P < 0.0001. The size of the node represents the p value of the log-rank test. Orange points represent favorable factors for DFS. Blue points represent risk factors for DFS. ∗P < 0.05, ∗∗P < 0.01, and ∗∗∗P < 0.001. Supplemental Figure 3: (A–H) consensus matrixes of PCa samples for each k (k = 2–7) from meta cohort based on expression abundance of 20 DMM regulators, displaying the clustering stability using 1000 iterations of hierarchical clustering. (H) Principal component analysis (PCA) of meta cohort based on expression abundance of 20 DMM regulators. Supplemental Figure 4: the correlation between ssGSEA score of 41 metabolic pathways and expression abundance of 20 DMM regulators in meta cohort and TCGA cohort. Supplemental Figure 5: (A–H) consensus matrixes of PCa samples for each k (k = 2–7) from meta cohort based on expression abundance of 337 DNA methylation-related prognostic genes, displaying the clustering stability using 1000 iterations of hierarchical clustering. Supplemental Figure 6: the relationship between DMS, tumor metabolism, and DNA methylation statue. (A) The heat map of the active level of metabolic pathways for three DNA methylation gene clusters in meta cohort. (B) The heat map of DNA methylation level (variance top 500 genes) for low and high DMS group in TCGA cohort. (C) Boxplot of average DNA methylation level (variance top 500 [file 2408637.f1.zip › Supplemental Figure.zip!/Supplemental Figure/Supplemental Figure 4.pdf]

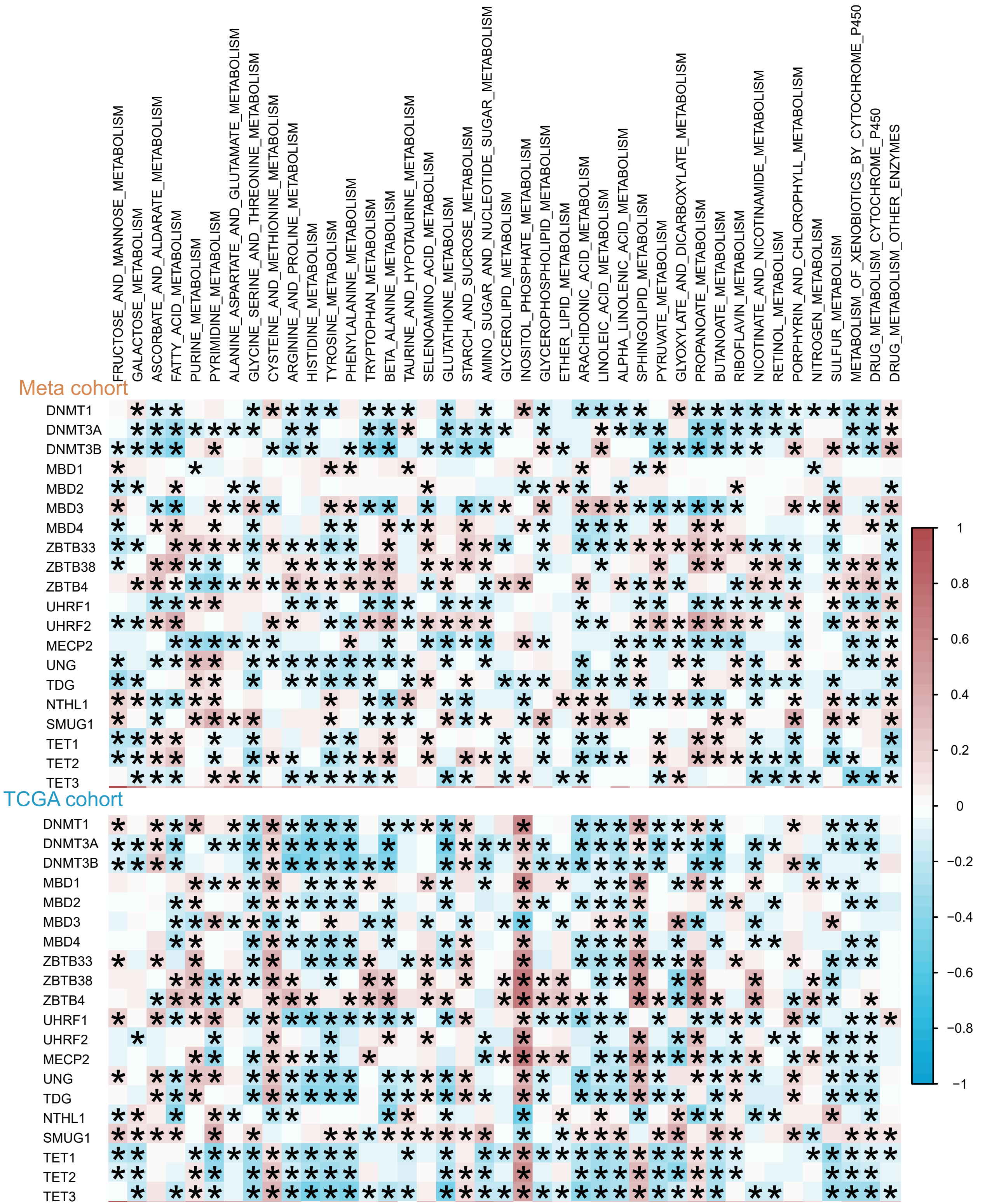
FRUCTOSE_AND_MANNOSE_METABOLISM
GALACTOSE_METABOLISM
ASCORBATE_AND_ALDARATE_METABOLISM
FATTY_ACID_METABOLISM
PURINE_METABOLISM
PYRIMIDINE_METABOLISM
ALANINE_ASPARTATE_AND_GLUTAMATE_METABOLISM
GLYCINE_SERINE_AND_THREONINE_METABOLISM
CYSTEINE_AND_METHIONINE_METABOLISM
ARGININE_AND_PROLINE_METABOLISM
HISTIDINE_METABOLISM
TYROSINE_METABOLISM
PHENYLALANINE_METABOLISM
TRYPTOPHAN_METABOLISM
BETA_ALANINE_METABOLISM
TAURINE_AND_HYPOTAURINE_METABOLISM
SELENOAMINO_ACID_METABOLISM
GLUTATHIONE_METABOLISM
STARCH_AND_SUCROSE_METABOLISM
AMINO_SUGAR_AND_NUCLEOTIDE_SUGAR_METABOLISM
GLYCEROLIPID_METABOLISM
INOSITOL_PHOSPHATE_METABOLISM
GLYCEROPHOSPHOLIPID_METABOLISM
ETHER_LIPID_METABOLISM
ARACHIDONIC_ACID_METABOLISM
LINOLEIC_ACID_METABOLISM
ALPHA_LINOLENIC_ACID_METABOLISM
SPHINGOLIPID_METABOLISM
PYRUVATE_METABOLISM
GLYOXYLATE_AND_DICARBOXYLATE_METABOLISM
PROPANOATE_METABOLISM
BUTANOATE_METABOLISM
RIBOFLAVIN_METABOLISM
NICOTINATE_AND_NICOTINAMIDE_METABOLISM
RETINOL_METABOLISM
PORPHYRIN_AND_CHLOROPHYLL_METABOLISM
NITROGEN_METABOLISM
SULFUR_METABOLISM
METABOLISM_OF_XENOBIOTICS_BY_CYTOCHROME_P450
DRUG_METABOLISM_CYTOCHROME_P450
DRUG_METABOLISM_OTHER_ENZYMES
Meta cohort
DNMT1
DNMT3A
DNMT3B
MBD1
MBD2
MBD3
MBD4
ZBTB33
ZBTB38
ZBTB4
UHRF1
UHRF2
MECP2
UNG
TDG
NTHL1
SMUG1
TET1
TET2
TET3
TCGA cohort
DNMT1
DNMT3A
DNMT3B
MBD1
MBD2
MBD3
MBD4
ZBTB33
ZBTB38
ZBTB4
UHRF1
UHRF2
MECP2
UNG
TDG
NTHL1
SMUG1
TET1
TET2
TET3
1
0.8
0.6
0.4
0.2
0
−0.2
−0.4
−0.6
−0.8
−1
