# Supplementary material for: DNA Methylation Modification Regulator-Mediated Molecular Clusters and Tumor Metabolic Characterization in Prostate Cancer
Source: J Oncol. 2021 Nov 11;2021:2408637. doi: 10.1155/2021/2408637 (PMC8601836; doi:10.1155/2021/2408637)
Supplement: Supplementary Materials — Supplemental Figure 1: (A) overview of study design. (B) Principal component analysis (PCA) of mRNA expression data of sample from five cohorts before batch correction. (C) Principal component analysis (PCA) of mRNA expression data of sample from five cohorts after batch correction. Supplemental Figure 2: (A) the volcano plot for correlation analysis between DNA methylation and mRNA expression of 20 DMM regulators. (B) Correlations and prognosis of DMM regulators in PCa patients in TCGA PRAD cohort. The red line represents a positive correlation with P < 0.0001, and the blue line represents a negative correlation with P < 0.0001. The size of the node represents the p value of the log-rank test. Orange points represent favorable factors for DFS. Blue points represent risk factors for DFS. ∗P < 0.05, ∗∗P < 0.01, and ∗∗∗P < 0.001. Supplemental Figure 3: (A–H) consensus matrixes of PCa samples for each k (k = 2–7) from meta cohort based on expression abundance of 20 DMM regulators, displaying the clustering stability using 1000 iterations of hierarchical clustering. (H) Principal component analysis (PCA) of meta cohort based on expression abundance of 20 DMM regulators. Supplemental Figure 4: the correlation between ssGSEA score of 41 metabolic pathways and expression abundance of 20 DMM regulators in meta cohort and TCGA cohort. Supplemental Figure 5: (A–H) consensus matrixes of PCa samples for each k (k = 2–7) from meta cohort based on expression abundance of 337 DNA methylation-related prognostic genes, displaying the clustering stability using 1000 iterations of hierarchical clustering. Supplemental Figure 6: the relationship between DMS, tumor metabolism, and DNA methylation statue. (A) The heat map of the active level of metabolic pathways for three DNA methylation gene clusters in meta cohort. (B) The heat map of DNA methylation level (variance top 500 genes) for low and high DMS group in TCGA cohort. (C) Boxplot of average DNA methylation level (variance top 500 [file 2408637.f1.zip › Supplemental Figure.zip!/Supplemental Figure/Supplemental Figure 6.pdf]

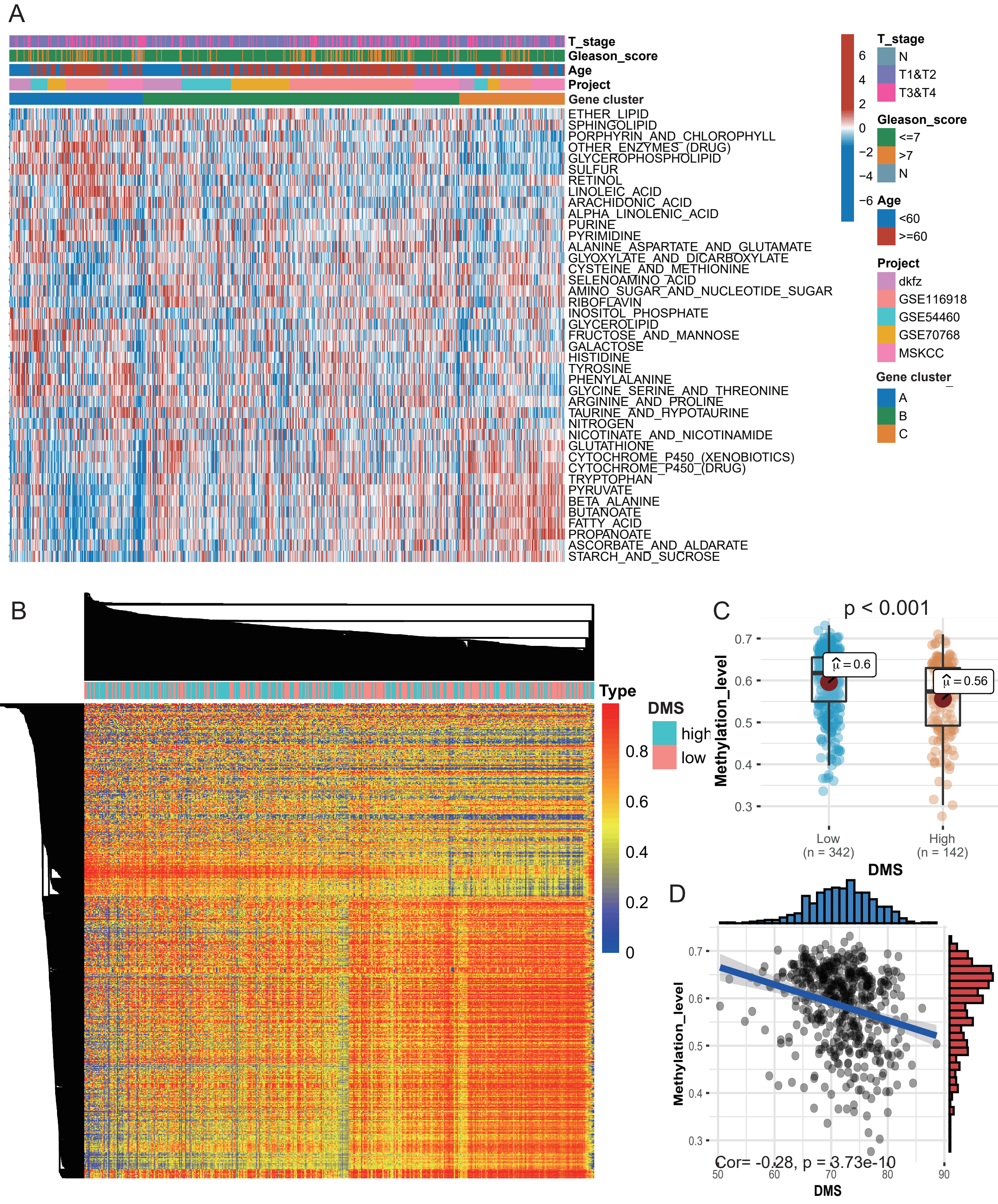
A
T_stage
Gleason_score
Age
Project
Gene cluster
ETHER_LIPID
SPHINGOLIPID
PORPHYRIN_AND_CHLOROPHYLL
OTHER_ENZYMES_(DRUG)
GLYCEROPHOSPHOLIPID
SULFUR
RETINOL
LINOLEIC_ACID
ARACHIDONIC_ACID
ALPHA_LINOLENIC_ACID
PURINE
PYRIMIDINE
ALANINE_ASPARTATE_AND_GLUTAMATE
GLYOXYLATE_AND_DICARBOXYLATE
CYSTEINE_AND_METHIONINE
SELENOAMINO_ACID
AMINO_SUGAR_AND_NUCLEOTIDE_SUGAR
RIBOFLAVIN
INOSITOL_PHOSPHATE
GLYCEROLIPID
FRUCTOSE_AND_MANNOSE
GALACTOSE
HISTIDINE
TYROSINE
PHENYLALANINE
GLYCINE_SERINE_AND_THREONINE
ARGININE_AND_PROLINE
TAURINE_AND_HYPOTAURINE
NITROGEN
NICOTINATE_AND_NICOTINAMIDE
GLUTATHIONE
CYTOCHROME_P450_(XENOBIOTICS)
CYTOCHROME_P450_(DRUG)
TRYPTOPHAN
PYRUVATE
BETA_ALANINE
BUTANOATE
FATTY_ACID
PROPANOATE
ASCORBATE_AND_ALDARATE
STARCH_AND_SUCROSE
6
4
2
0
−2
−4
−6
T_stage
N
T1&T2
T3&T4
Gleason_score
<=7
>7
N
Age
<60
>=60
Project
dkfz
GSE116918
GSE54460
GSE70768
MSKCC
Gene cluster
A
B
C
B
Type
DMS
high
low
0.8
0.6
0.4
0.2
0
C
p < 0.001
Methylation_level
0.7
0.6
0.5
0.4
0.3
μ̂ = 0.6
μ̂ = 0.56
Low
(n = 342)
High
(n = 142)
DMS
D
DMS
Methylation_level
0.7
0.6
0.5
0.4
0.3
Cor= -0.28, p = 3.73e-10
50
60
70
80
90
DMS
