# Supplementary material for: DNA Methylation Modification Regulator-Mediated Molecular Clusters and Tumor Metabolic Characterization in Prostate Cancer
Source: J Oncol. 2021 Nov 11;2021:2408637. doi: 10.1155/2021/2408637 (PMC8601836; doi:10.1155/2021/2408637)
Supplement: Supplementary Materials — Supplemental Figure 1: (A) overview of study design. (B) Principal component analysis (PCA) of mRNA expression data of sample from five cohorts before batch correction. (C) Principal component analysis (PCA) of mRNA expression data of sample from five cohorts after batch correction. Supplemental Figure 2: (A) the volcano plot for correlation analysis between DNA methylation and mRNA expression of 20 DMM regulators. (B) Correlations and prognosis of DMM regulators in PCa patients in TCGA PRAD cohort. The red line represents a positive correlation with P < 0.0001, and the blue line represents a negative correlation with P < 0.0001. The size of the node represents the p value of the log-rank test. Orange points represent favorable factors for DFS. Blue points represent risk factors for DFS. ∗P < 0.05, ∗∗P < 0.01, and ∗∗∗P < 0.001. Supplemental Figure 3: (A–H) consensus matrixes of PCa samples for each k (k = 2–7) from meta cohort based on expression abundance of 20 DMM regulators, displaying the clustering stability using 1000 iterations of hierarchical clustering. (H) Principal component analysis (PCA) of meta cohort based on expression abundance of 20 DMM regulators. Supplemental Figure 4: the correlation between ssGSEA score of 41 metabolic pathways and expression abundance of 20 DMM regulators in meta cohort and TCGA cohort. Supplemental Figure 5: (A–H) consensus matrixes of PCa samples for each k (k = 2–7) from meta cohort based on expression abundance of 337 DNA methylation-related prognostic genes, displaying the clustering stability using 1000 iterations of hierarchical clustering. Supplemental Figure 6: the relationship between DMS, tumor metabolism, and DNA methylation statue. (A) The heat map of the active level of metabolic pathways for three DNA methylation gene clusters in meta cohort. (B) The heat map of DNA methylation level (variance top 500 genes) for low and high DMS group in TCGA cohort. (C) Boxplot of average DNA methylation level (variance top 500 [file 2408637.f1.zip › Supplemental Figure.zip!/Supplemental Figure/Supplemental Figure 7.pdf]

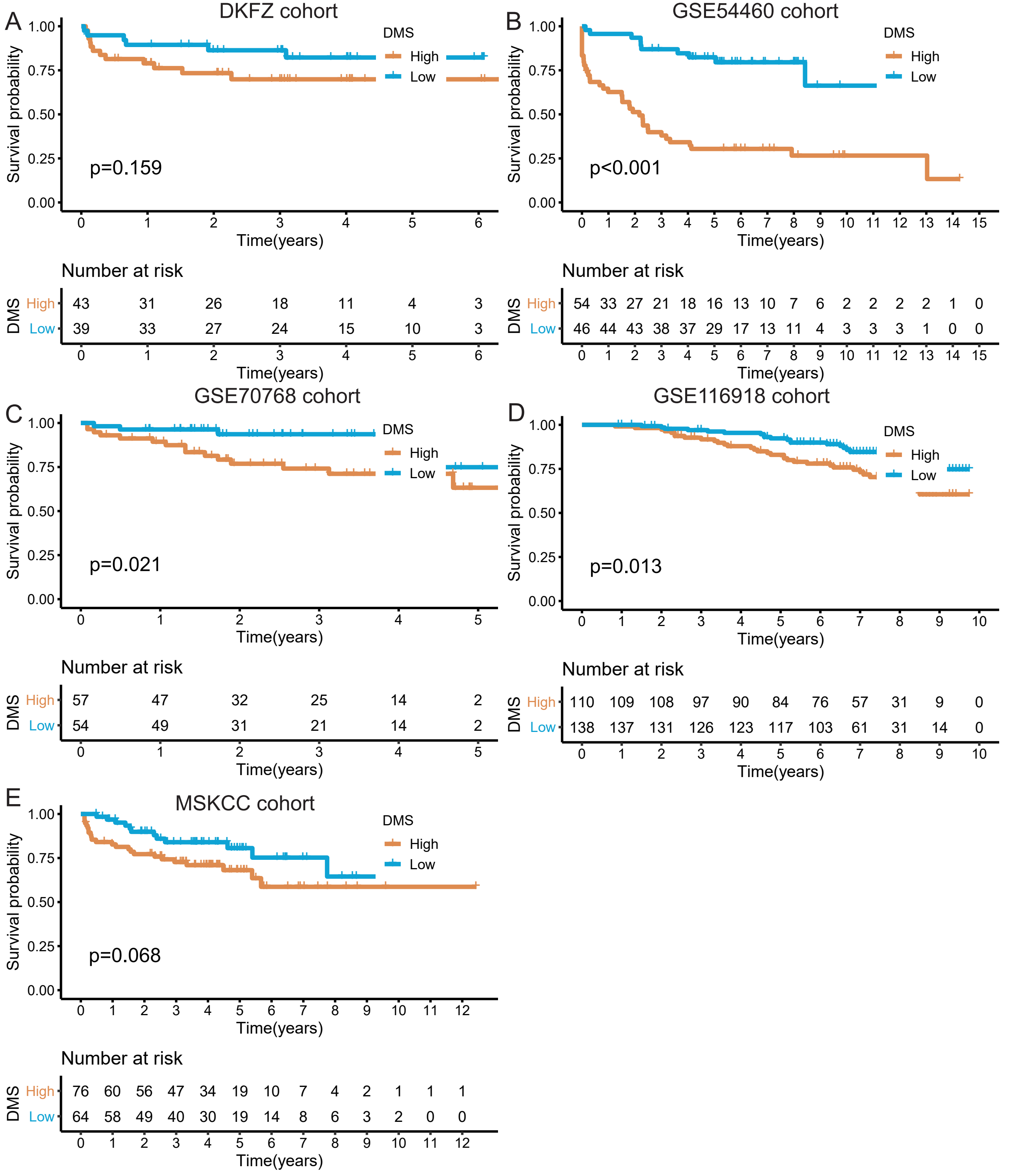
A
DKFZ cohort
Survival probability
1.00
0.75
0.50
0.25
0.00
DMS
High
Low
p=0.159
0 1 2 3 4 5 6
Time(years)
Number at risk
DMS
High 43 31 26 18 11 4 3
Low 39 33 27 24 15 10 3
0 1 2 3 4 5 6
Time(years)
B
GSE54460 cohort
Survival probability
1.00
0.75
0.50
0.25
0.00
DMS
High
Low
p<0.001
0 1 2 3 4 5 6 7 8 9 10 11 12 13 14 15
Time(years)
Number at risk
DMS
High 54 33 27 21 18 16 13 10 7 6 2 2 2 2 1 0
Low 46 44 43 38 37 29 17 13 11 4 3 3 3 1 0 0
0 1 2 3 4 5 6 7 8 9 10 11 12 13 14 15
Time(years)
C
GSE70768 cohort
Survival probability
1.00
0.75
0.50
0.25
0.00
DMS
High
Low
p=0.021
0 1 2 3 4 5
Time(years)
Number at risk
DMS
High 57 47 32 25 14 2
Low 54 49 31 21 14 2
0 1 2 3 4 5
Time(years)
D
GSE116918 cohort
Survival probability
1.00
0.75
0.50
0.25
0.00
DMS
High
Low
p=0.013
0 1 2 3 4 5 6 7 8 9 10
Time(years)
Number at risk
DMS
High 110 109 108 97 90 84 76 57 31 9 0
Low 138 137 131 126 123 117 103 61 31 14 0
0 1 2 3 4 5 6 7 8 9 10
Time(years)
E
MSKCC cohort
Survival probability
1.00
0.75
0.50
0.25
0.00
DMS
High
Low
p=0.068
0 1 2 3 4 5 6 7 8 9 10 11 12
Time(years)
Number at risk
DMS
High 76 60 56 47 34 19 10 7 4 2 1 1 1
Low 64 58 49 40 30 19 14 8 6 3 2 0 0
0 1 2 3 4 5 6 7 8 9 10 11 12
Time(years)
