# Supplementary material for: DNA Methylation Modification Regulator-Mediated Molecular Clusters and Tumor Metabolic Characterization in Prostate Cancer
Source: J Oncol. 2021 Nov 11;2021:2408637. doi: 10.1155/2021/2408637 (PMC8601836; doi:10.1155/2021/2408637)
Supplement: Supplementary Materials — Supplemental Figure 1: (A) overview of study design. (B) Principal component analysis (PCA) of mRNA expression data of sample from five cohorts before batch correction. (C) Principal component analysis (PCA) of mRNA expression data of sample from five cohorts after batch correction. Supplemental Figure 2: (A) the volcano plot for correlation analysis between DNA methylation and mRNA expression of 20 DMM regulators. (B) Correlations and prognosis of DMM regulators in PCa patients in TCGA PRAD cohort. The red line represents a positive correlation with P < 0.0001, and the blue line represents a negative correlation with P < 0.0001. The size of the node represents the p value of the log-rank test. Orange points represent favorable factors for DFS. Blue points represent risk factors for DFS. ∗P < 0.05, ∗∗P < 0.01, and ∗∗∗P < 0.001. Supplemental Figure 3: (A–H) consensus matrixes of PCa samples for each k (k = 2–7) from meta cohort based on expression abundance of 20 DMM regulators, displaying the clustering stability using 1000 iterations of hierarchical clustering. (H) Principal component analysis (PCA) of meta cohort based on expression abundance of 20 DMM regulators. Supplemental Figure 4: the correlation between ssGSEA score of 41 metabolic pathways and expression abundance of 20 DMM regulators in meta cohort and TCGA cohort. Supplemental Figure 5: (A–H) consensus matrixes of PCa samples for each k (k = 2–7) from meta cohort based on expression abundance of 337 DNA methylation-related prognostic genes, displaying the clustering stability using 1000 iterations of hierarchical clustering. Supplemental Figure 6: the relationship between DMS, tumor metabolism, and DNA methylation statue. (A) The heat map of the active level of metabolic pathways for three DNA methylation gene clusters in meta cohort. (B) The heat map of DNA methylation level (variance top 500 genes) for low and high DMS group in TCGA cohort. (C) Boxplot of average DNA methylation level (variance top 500 [file 2408637.f1.zip › Supplemental Figure.zip!/Supplemental Figure/Supplemental Figure 8.pdf]

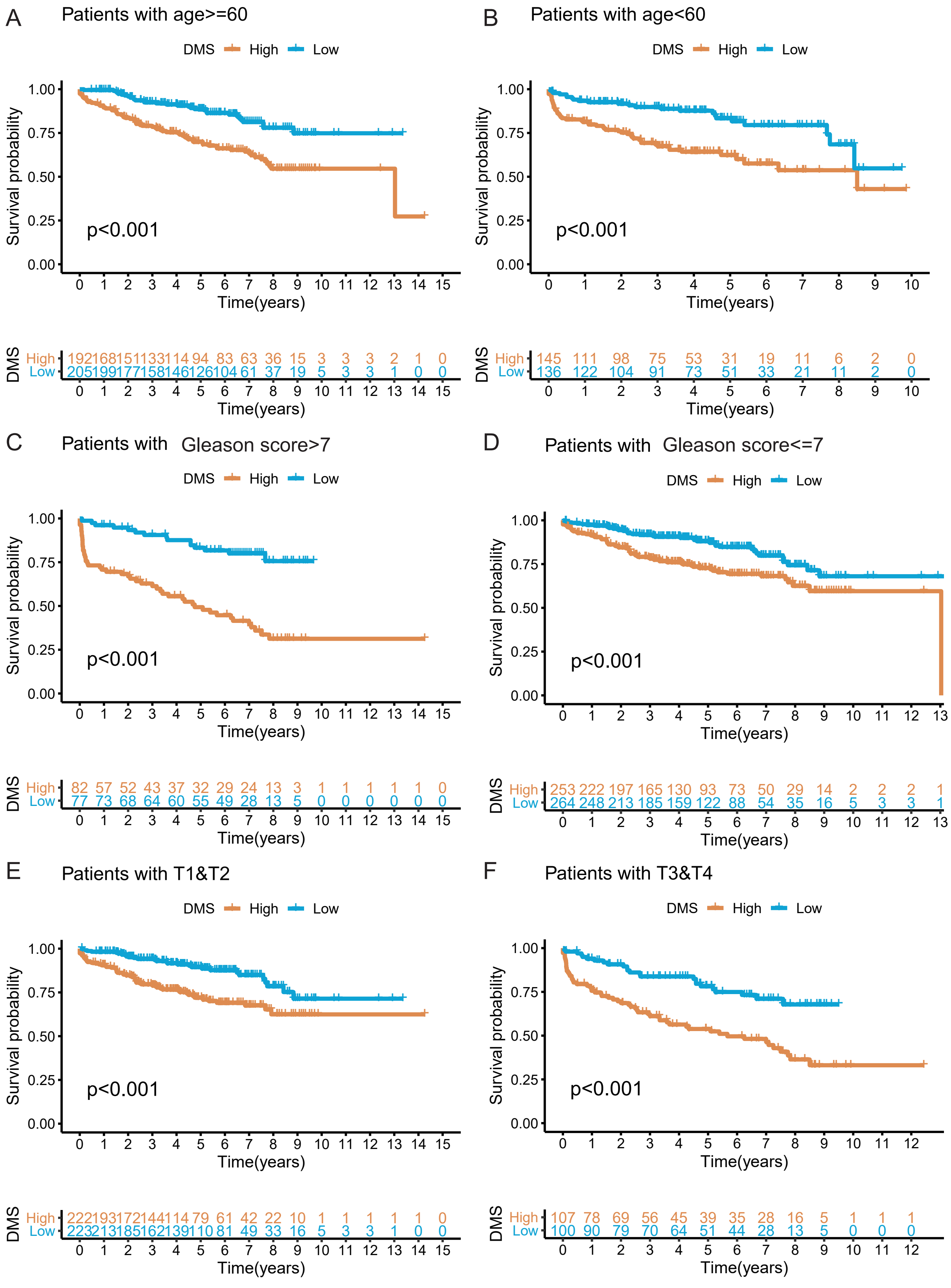
A
Patients with age>=60
DMS High Low
Survival probability
p<0.001
Time(years)
High 192 168 151 133 114 94 83 63 36 15 3 3 3 2 1 0
Low 205 199 177 158 146 126 104 61 37 19 5 3 3 1 0 0
B
Patients with age<60
DMS High Low
Survival probability
p<0.001
Time(years)
High 145 111 98 75 53 31 19 11 6 2 0
Low 136 122 104 91 73 51 33 21 11 2 0
C
Patients with Gleason score>7
DMS High Low
Survival probability
p<0.001
Time(years)
High 82 57 52 43 37 32 29 24 13 3 1 1 1 1 1 0
Low 77 73 68 64 60 55 49 28 13 5 0 0 0 0 0 0
D
Patients with Gleason score<=7
DMS High Low
Survival probability
p<0.001
Time(years)
High 253 222 197 165 130 93 73 50 29 14 2 2 2 1
Low 264 248 213 185 159 122 88 54 35 16 5 3 3 1
E
Patients with T1&T2
DMS High Low
Survival probability
p<0.001
Time(years)
High 222 193 172 144 114 79 61 42 22 10 1 1 1 1 1 0
Low 223 213 185 162 139 110 81 49 33 16 5 3 3 1 0 0
F
Patients with T3&T4
DMS High Low
Survival probability
p<0.001
Time(years)
High 107 78 69 56 45 39 35 28 16 5 1 1 1
Low 100 90 79 70 64 51 44 28 13 5 0 0 0
