# Supplementary material for: DNA Methylation Modification Regulator-Mediated Molecular Clusters and Tumor Metabolic Characterization in Prostate Cancer
Source: J Oncol. 2021 Nov 11;2021:2408637. doi: 10.1155/2021/2408637 (PMC8601836; doi:10.1155/2021/2408637)
Supplement: Supplementary Materials — Supplemental Figure 1: (A) overview of study design. (B) Principal component analysis (PCA) of mRNA expression data of sample from five cohorts before batch correction. (C) Principal component analysis (PCA) of mRNA expression data of sample from five cohorts after batch correction. Supplemental Figure 2: (A) the volcano plot for correlation analysis between DNA methylation and mRNA expression of 20 DMM regulators. (B) Correlations and prognosis of DMM regulators in PCa patients in TCGA PRAD cohort. The red line represents a positive correlation with P < 0.0001, and the blue line represents a negative correlation with P < 0.0001. The size of the node represents the p value of the log-rank test. Orange points represent favorable factors for DFS. Blue points represent risk factors for DFS. ∗P < 0.05, ∗∗P < 0.01, and ∗∗∗P < 0.001. Supplemental Figure 3: (A–H) consensus matrixes of PCa samples for each k (k = 2–7) from meta cohort based on expression abundance of 20 DMM regulators, displaying the clustering stability using 1000 iterations of hierarchical clustering. (H) Principal component analysis (PCA) of meta cohort based on expression abundance of 20 DMM regulators. Supplemental Figure 4: the correlation between ssGSEA score of 41 metabolic pathways and expression abundance of 20 DMM regulators in meta cohort and TCGA cohort. Supplemental Figure 5: (A–H) consensus matrixes of PCa samples for each k (k = 2–7) from meta cohort based on expression abundance of 337 DNA methylation-related prognostic genes, displaying the clustering stability using 1000 iterations of hierarchical clustering. Supplemental Figure 6: the relationship between DMS, tumor metabolism, and DNA methylation statue. (A) The heat map of the active level of metabolic pathways for three DNA methylation gene clusters in meta cohort. (B) The heat map of DNA methylation level (variance top 500 genes) for low and high DMS group in TCGA cohort. (C) Boxplot of average DNA methylation level (variance top 500 [file 2408637.f1.zip › Supplemental Figure.zip!/Supplemental Figure/Supplemental Figure 9.pdf]

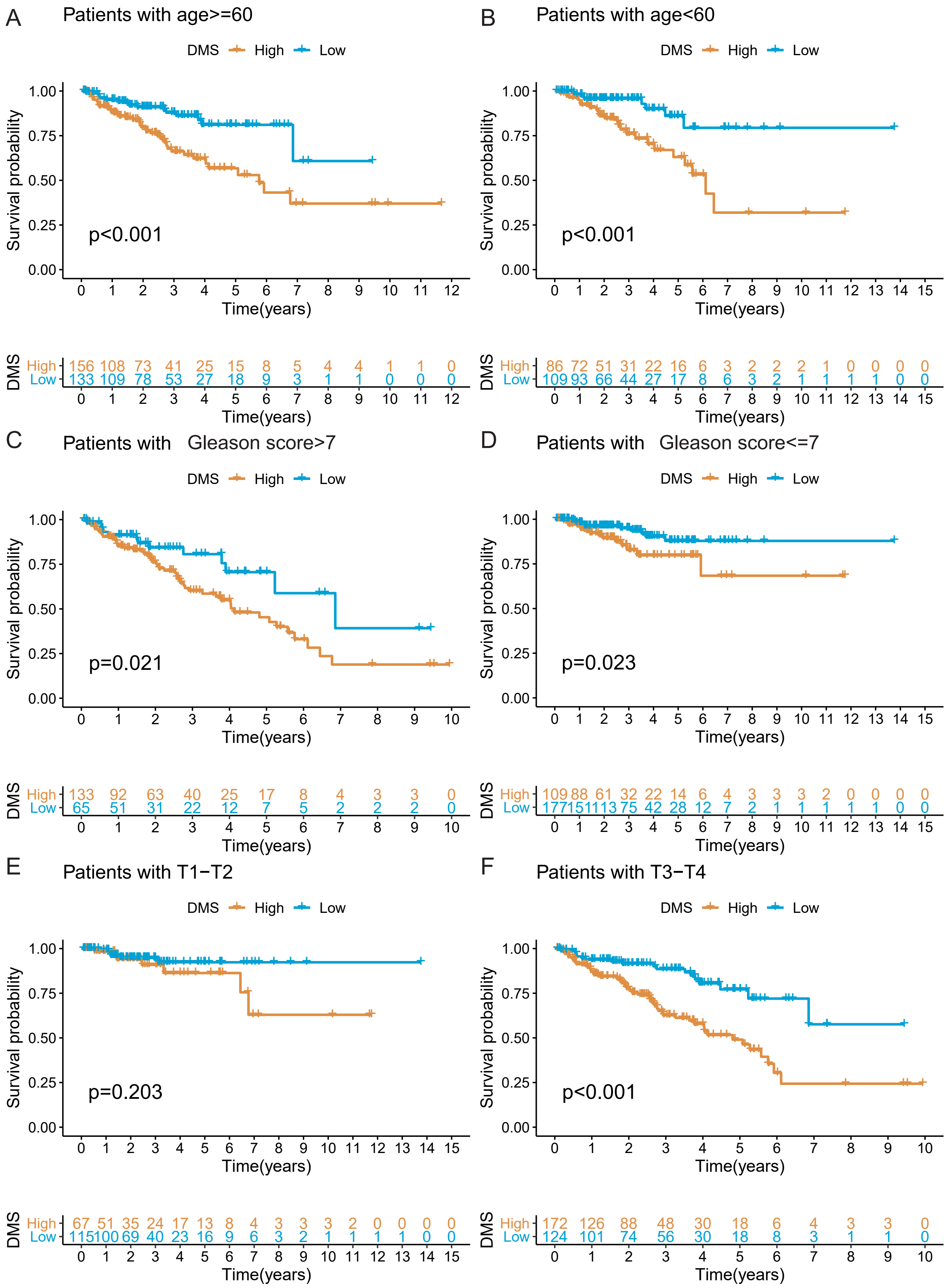
A
Patients with age>=60
DMS High Low
Survival probability
1.00
0.75
0.50
0.25
0.00
p<0.001
0 1 2 3 4 5 6 7 8 9 10 11 12
Time(years)
DMS
High 156 108 73 41 25 15 8 5 4 4 1 1 0
Low 133 109 78 53 27 18 9 3 1 1 0 0 0
0 1 2 3 4 5 6 7 8 9 10 11 12
Time(years)
B
Patients with age<60
DMS High Low
Survival probability
1.00
0.75
0.50
0.25
0.00
p<0.001
0 1 2 3 4 5 6 7 8 9 10 11 12 13 14 15
Time(years)
DMS
High 86 72 51 31 22 16 6 3 2 2 2 1 0 0 0 0
Low 109 93 66 44 27 17 8 6 3 2 1 1 1 1 0 0
0 1 2 3 4 5 6 7 8 9 10 11 12 13 14 15
Time(years)
C
Patients with Gleason score>7
DMS High Low
Survival probability
1.00
0.75
0.50
0.25
0.00
p=0.021
0 1 2 3 4 5 6 7 8 9 10
Time(years)
DMS
High 133 92 63 40 25 17 8 4 3 3 0
Low 65 51 31 22 12 7 5 2 2 2 0
0 1 2 3 4 5 6 7 8 9 10
Time(years)
D
Patients with Gleason score<=7
DMS High Low
Survival probability
1.00
0.75
0.50
0.25
0.00
p=0.023
0 1 2 3 4 5 6 7 8 9 10 11 12 13 14 15
Time(years)
DMS
High 109 88 61 32 22 14 6 4 3 3 3 2 0 0 0 0
Low 177151113 75 42 28 12 7 2 1 1 1 1 1 0 0
0 1 2 3 4 5 6 7 8 9 10 11 12 13 14 15
Time(years)
E
Patients with T1−T2
DMS High Low
Survival probability
1.00
0.75
0.50
0.25
0.00
p=0.203
0 1 2 3 4 5 6 7 8 9 10 11 12 13 14 15
Time(years)
DMS
High 67 51 35 24 17 13 8 4 3 3 3 2 0 0 0 0
Low 115100 69 40 23 16 9 6 3 2 1 1 1 1 0 0
0 1 2 3 4 5 6 7 8 9 10 11 12 13 14 15
Time(years)
F
Patients with T3−T4
DMS High Low
Survival probability
1.00
0.75
0.50
0.25
0.00
p<0.001
0 1 2 3 4 5 6 7 8 9 10
Time(years)
DMS
High 172 126 88 48 30 18 6 4 3 3 0
Low 124 101 74 56 30 18 8 3 1 1 0
0 1 2 3 4 5 6 7 8 9 10
Time(years)
